# Enhancing Super‐Resolution Spatial Transcriptomics Data by Transfer Learning

**DOI:** 10.1002/advs.76601

**Published:** 2026-07-20

**Authors:** Xiaoyu Li, Lihua Zhang, Wenwen Min

**Affiliations:** ^1^ School of Artificial Intelligence Wuhan University Wuhan China; ^2^ School of Computer Science Yunnan University Kunming China

**Keywords:** graph neural network, spatial transcriptomics, super‐resolution, transfer learning

## Abstract

High‐definition spatial transcriptomics (ST) technologies such as Visium HD enable subcellular tissue characterization but remain constrained by their limited accessibility due to high costs and technical complexity. Existing super‐resolution methods predominantly rely on an image‐guided paradigm, premised on the assumption that gene expression strictly mirrors histological morphology. However, this assumption breaks down for genes with complex spatial distributions lacking distinct visual correlates, often leading to biological artifacts. To address this, we introduce SpotZoomer, a framework that formulates resolution enhancement as a knowledge transfer problem via generative domain adaptation. It leverages public high‐definition ST data as a “teacher” to learn intrinsic spatial expression priors, which are then transferred to coarse spot data to reconstruct high‐fidelity gene profiles that capture molecular details beyond the reach of morphological guidance alone. Extensive benchmarking across 19 datasets demonstrates the substantial value of the reference‐based paradigm implemented by SpotZoomer over the reference‐free image‐only paradigm, achieving improved reconstruction accuracy and biological fidelity while complementing rather than displacing reference‐free methods in settings where high‐resolution priors are unavailable. SpotZoomer thus provides a scalable, data‐driven strategy for upgrading standard ST resources to subcellular resolution.

## Graphical abstract

SpotZoomer employs a transfer‐learning‐based strategy to enhance the resolution of Visium data by leveraging available high‐resolution priors. The resulting super‐resolved maps enable sharper delineation of cell boundaries and more precise inference of cell–cell communication patterns that would otherwise remain obscured at native resolution.

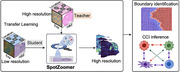

## 1 Introduction

Spatial transcriptomics (ST) has made it possible to study gene expression within intact tissue architecture, enabling the precise mapping of intercellular communication [1] and tumor microenvironment heterogeneity [2, 3, 4, 5]. Currently, the field faces a dichotomy between coverage and resolution. On one hand, widely adopted spot‐level platforms (e.g., 10x Genomics Visium) offer robust whole‐transcriptome coverage but suffer from coarse spatial resolution (55 µm diameter) [6, 7, 8, 9], where a single spot inevitably mixes transcriptomes from multiple cells [10, 11]. On the other hand, emerging high‐definition technologies, such as Xenium [12] and the newly released Visium HD [13], have pushed the limit to subcellular scales (2 µm bin size), resolving the trade‐off between resolution and coverage [14, 15]. However, the prohibitive cost and technical complexity of these next‐generation platforms restrict their widespread adoption [16]. As a result, many existing Visium datasets contain rich molecular information but lack the spatial granularity needed to resolve cell‐level tissue organization. This creates a need for computational methods that can enhance standard‐resolution ST data while preserving biological fidelity.

A growing body of work has shown that histological morphology is informative for spatial gene expression, motivating computational approaches that infer molecular patterns from H&E images [17, 18, 19]. Building upon this observation, several reference‐free approaches have been proposed to infer gene expression patterns directly from histological images. Methods such as XFuse [20] and TESLA [21] leverage histological images (H&E) to infer higher‐resolution expression patterns via deep generative models or physical super‐pixel networks. More recently, iStar [22] and scstGCN [23] introduced a multi‐scale vision backbone to capture local and global image features for expression prediction. These methods have advanced the field, but they mainly frame super‐resolution as a morphology‐to‐expression prediction problem. This can be limiting because not all transcriptional programs have clear histological correlates, and sparse or context‐specific genes may be poorly recovered from visual texture alone. Moreover, reference‐free models do not directly use the spatial expression distributions observed in true high‐resolution ST data, even when such data are available for related tissues or platforms.

Here, we present SpotZoomer, a reference‐guided framework for reconstructing high‐resolution spatial transcriptomic profiles from standard Visium data. Rather than relying only on histological appearance, SpotZoomer uses high‐resolution ST data, such as Visium HD, as a teacher domain from which it learns fine‐scale expression structure. These learned priors are then transferred to a lower‐resolution student domain, allowing the model to infer spatially detailed expression maps from Visium data while being constrained by both molecular and morphological information. To validate this approach, we performed a comprehensive benchmarking study across 19 datasets spanning diverse species and tissue types. Our results demonstrate that SpotZoomer significantly outperforms existing super‐resolution methods (including iStar and scstGCN), achieving superior accuracy in recovering single‐cell expression profiles. Furthermore, we applied SpotZoomer to colorectal cancer and lung fibrosis datasets, where it successfully recovered fine‐grained tissue architectures [24, 25] and unmasked specific tumor‐associated microenvironments [26] that were obscured at native resolution. Together, these results suggest that reference‐guided super‐resolution can make high‐definition spatial analysis more accessible for existing and future spot‐level ST datasets.

## 2 Results

### 2.1 Overview of SpotZoomer

**FIGURE 1:**
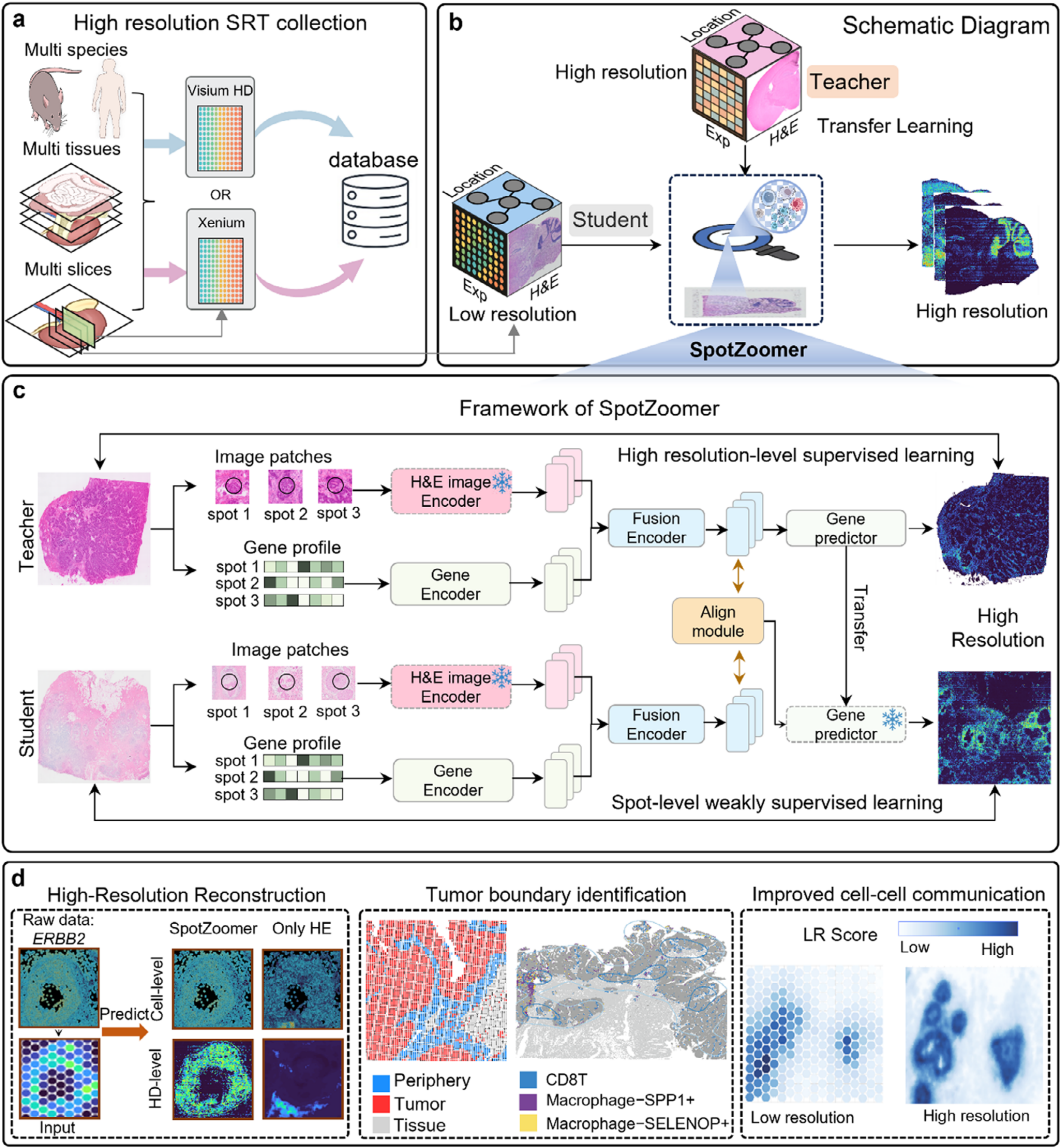
Overview of SpotZoomer. (a) High resolution ST database. We collected high‐resolution ST data from multiple mouse and human tissues obtained from the Visium HD and Xenium platforms as reference data. (b) Pipeline of SpotZoomer. SpotZoomer takes low‐resolution Visium data and the reference data as inputs and generates super‐resolution gene expression profiles for the Visium data via transfer learning. The reference data serves as the teacher, and the Visium data acts as the student. (c) SpotZoomer first generates pseudo low‐resolution ST data from the reference data by aggregating gene expression values from neighboring spots. It then employs an H&E image encoder and a gene encoder to extract features from the morphological images and gene expression profiles of the (pseudo) low‐resolution data. Next, SpotZoomer adopts a fusion encoder with an attention mechanism to generate a fused representation by integrating gene expression and morphological image features. After that, a gene predictor module is applied to the fused representation to predict high‐resolution expression values for the teacher. To make the parameters of the gene predictor module applicable to low‐resolution ST data (student), we leverage an align module to align the fused representations from the teacher and student. Finally, the prediction encoder is constrained with spot‐level weak self‐supervised learning and used to predict super‐resolution gene expression levels for the low‐resolution ST data. Snowflake icons indicate modules whose weights are frozen at the depicted stage: the H&E image encoder (UNI) is loaded with publicly released pre‐trained weights and kept frozen throughout training, while the snowflake on the Student‐row Gene predictor indicates that, during the spot‐level weakly supervised fine‐tuning phase, only its final layer is updated and the remaining parameters are kept frozen. (d) Applications of SpotZoomer include accurate reconstruction of super‐resolution data, identification of tumor boundaries, and uncovering sophisticated cellular communication.

SpotZoomer treats the available high‐resolution spatial transcriptomics data from Visium HD or Xenium platforms as reference data (Figure 1a). It is a transfer learning‐based model designed to enhance the spatial resolution of Visium data using the prior knowledge acquired from the reference data (Figure 1b). The model consists of four key stages (Figure 1c and Figure S1). First, SpotZoomer generates pseudo low‐resolution ST data from reference data by aggregating gene expression values from neighboring spots. Then, transcriptomic and morphological features are extracted using a unified extraction methodology for (pseudo) low‐resolution data (Figure S1a). Gene expression profiles are encoded using a graph contrastive learning framework to generate latent transcriptomic representations, and histological features are derived from H&E‐stained tissue sections using a pretrained large‐scale pathology foundation model. Second, the two modalities are integrated via an attention‐based fusion module to produce a joint multimodal representation (Figure S1b). A gene predictor module is applied to the joint representation to predict high‐resolution expression values for the reference data. Third, to make the parameters of the gene predictor module applicable to low‐resolution ST data, we leverage an alignment module to align the fused representations from pseudo low‐resolution data and low‐resolution data [27]. Specifically, the latent representations from pseudo low‐ and low‐resolution data are aligned using a triplet loss function [28, 29] (Figure S1c). Fourth, the prediction encoder is constrained with spot‐level weak self‐supervised learning and used to generate super‐resolution gene expression levels for the low‐resolution ST data (Figure S1d). The generated super‐resolution data can be used to identify tumor boundaries, cellular microenvironments, and previously unresolvable cell types and cell–cell communication patterns in low‐resolution expression maps (Figure 1d).

### 2.2 SpotZoomer Achieves High‐Fidelity Structural Restoration by Teacher‐Student Domain Adaptation

To comprehensively evaluate the effectiveness of SpotZoomer in generating super‐resolution gene expression values, we conducted experiments on spatial transcriptomics datasets obtained from different platforms, including Visium HD, Xenium, and Visium. We compared SpotZoomer with four existing methods designed for super‐resolution gene expression prediction: XFuse [20], TESLA [21], iStar [22], and scstGCN [23] (**Methods: “Comparative methods”**). These methods utilize histological images to enhance the resolution of spatial transcriptomics data. For the high‐resolution datasets (i.e., Visium HD and Xenium), we generated pseudo low‐resolution data at the Visium level and subsequently applied each method to reconstruct the corresponding high‐resolution gene expression profiles. The predicted results were then compared with the original high‐resolution data, which serve as the ground truth, using several quantitative metrics (**Methods**: “**Evaluation metrics**”). For the Visium datasets, we generated pseudo low‐resolution inputs by aggregating gene expression values from neighboring spots. The performance of each method was evaluated by comparing the reconstructed gene expression profiles with the original Visium data (**Methods: “Data preprocessing”**).

**FIGURE 2:**
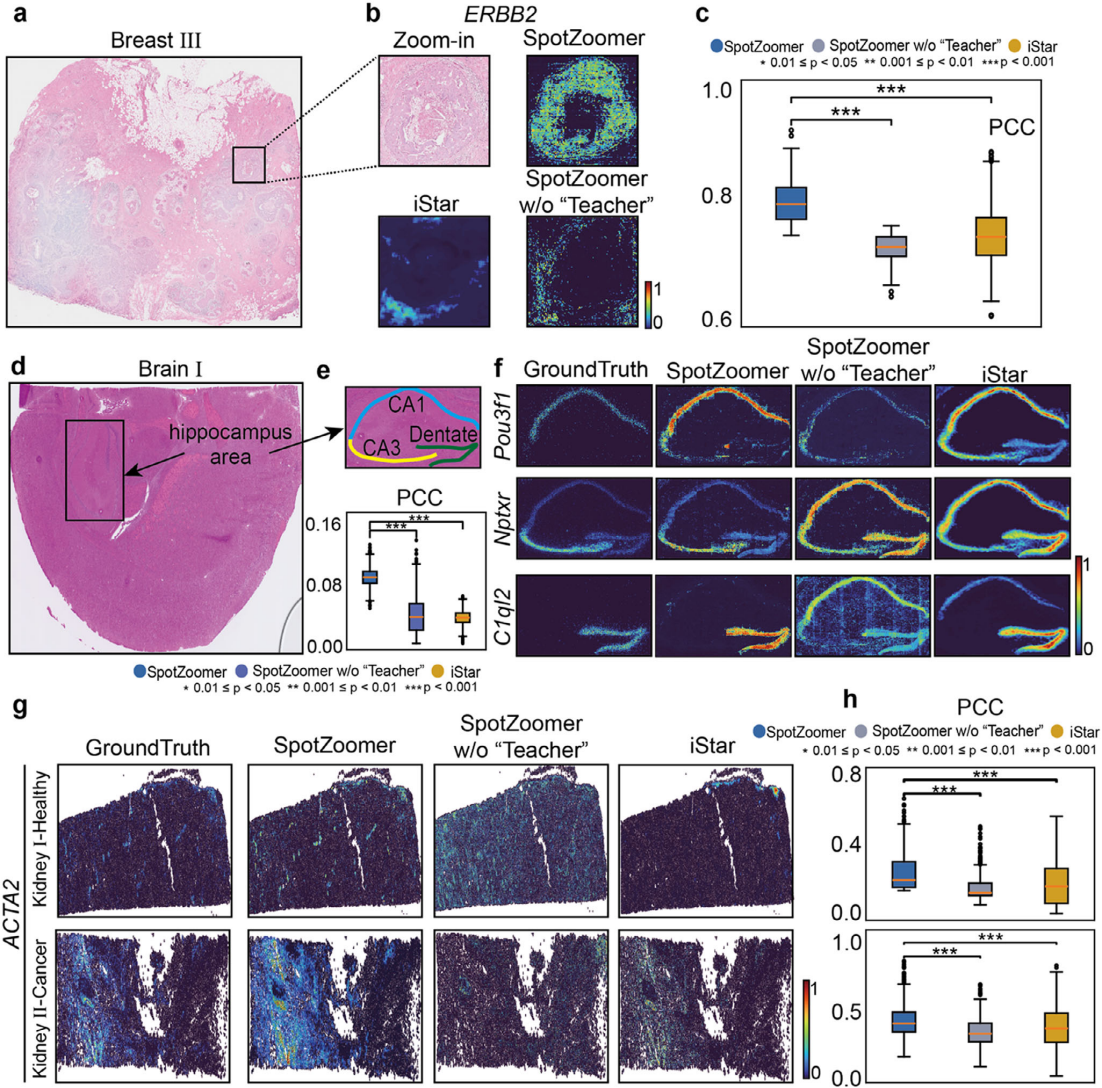
The high‐definition "Teacher" prior is essential for in silico re‐sequencing and structural restoration. (a–c) Ablation study on single‐cell resolution recovery in human breast cancer (Breast III). (a) H&E reference showing the tumor region. (b) Visual comparison of ERBB2 expression. SpotZoomer accurately reconstructs the ring‐like tumor structure consistent with the zoomed‐in H&E, whereas removing the high‐definition guidance (SpotZoomer w/o "Teacher") leads to signal degradation and loss of cellular definition, similar to the baseline (iStar). (c) Quantitative evaluation (PCC) confirms that the full SpotZoomer model significantly outperforms the ablated version and iStar (^***^ *p* < 0.001). (d–f) Validation of anatomical structure recovery in the mouse brain hippocampus (Brain I). (d) H&E reference. (e) Top: Schematic of hippocampal sub‐regions (CA1, CA3, Dentate Gyrus). Bottom: Box plot of PCC performance. (f) Visualization of layer‐specific marker genes (Pou3f1 for CA1, Nptxr for CA3, C1ql2 for Dentate Gyrus). SpotZoomer faithfully restores the sharp, continuous laminar boundaries. Without the "Teacher" prior, the model fails to define these specific anatomical shapes, resulting in fragmented patterns. (g–h) Characterization of disease‐specific heterogeneity in human kidney tissues. (g) Visualization of the fibroblast marker ACTA2 in healthy (Kidney I) and cancer (Kidney II) states. SpotZoomer captures the biological shift from sparse, scattered expression (Healthy) to dense, structural accumulation (Cancer). (h) Quantitative benchmarking (PCC) affirms that the "Teacher" mechanism ensures superior fidelity across biological conditions compared to the ablated model and iStar. Physical dimensions of displayed fields of view: (a) Breast III full‐tissue H&E corresponds to approximately 6.5 × 6.5 mm; (b) ERBB2 zoom‐in panels correspond to approximately 800 × 800 µm; (d) Brain I full‐tissue H&E corresponds to approximately 6.5 × 6.5 mm; (e) hippocampus area zoom corresponds to approximately 2 × 1.5 mm; (f) gene expression panels (Pou3f1, Nptxr, C1ql2) correspond to approximately 2 × 1.5 mm each; (g) kidney comparison panels (ACTA2 on Kidney I‐Healthy and Kidney II‐Cancer) correspond to approximately 6 × 4 mm each.

To decipher the mechanism underlying SpotZoomer's superior performance, we conducted an ablation study to evaluate the necessity of the high‐definition “Teacher” domain. Visual inspection of the marker gene *ERBB2* revealed that the full SpotZoomer model accurately reconstructed the distribution of the tumor, resolving the ring‐like architecture with fidelity comparable to the histological reference (Figure 2b). When the teacher guidance is removed, SpotZoomer suffers a substantial performance collapse, generating blurred artifacts that closely resemble those of the image‐guided baseline (iStar). This performance degradation is quantitatively validated by a marked reduction in the Pearson correlation coefficient (PCC) (Figure 2c), demonstrating that histological images alone are inadequate for reconstructing high‐definition details.

We further investigated whether this data‐driven prior is essential for defining complex anatomical structures. In the mouse hippocampus (Figure 2d), distinct sub‐regions, including CA1, CA3, and the Dentate Gyrus, are characterized by spatially restricted marker genes, namely *Pou3f1*, *Nptxr*, and *C1ql2*, respectively (Figure 2f). SpotZoomer faithfully restores sharp and continuous laminar boundaries across these regions. In contrast, the ablated model fails to delineate the characteristic shape of the Dentate Gyrus and instead produces fragmented and nonspecific signals. This loss of structural integrity is further supported by quantitative evaluation, in which the full model achieves significantly higher accuracy (Figure 2e). Collectively, these results demonstrate that intrinsic spatial priors learned from the Teacher domain are crucial for preventing structural hallucinations.

Finally, we assessed the robustness of the model in capturing disease‐related heterogeneity using human kidney tissues. Visualization of the fibroblast marker *ACTA2* revealed a clear shift in biological state, from sparse and scattered expression in healthy tissue (Kidney I) to dense and spatially organized accumulation in cancer tissue (Kidney II) (Figure 2g). SpotZoomer successfully captured this contrast while preserving the biological fidelity of the tumor microenvironment. In comparison, models without histology‐driven priors or those relying solely on image guidance struggled to distinguish these condition‐specific patterns and frequently over‐smoothed sparse signals in healthy regions. Quantitative benchmarking further confirmed that SpotZoomer consistently maintains high fidelity across diverse biological conditions (Figure 2h), validating the Teacher‐Student paradigm as a robust framework for enhancing spatial transcriptomics.

Following the successful validation on breast and brain tissues, we sought to establish the universality of SpotZoomer across a broader spectrum of biological contexts. We broadened our benchmarking to include 19 datasets spanning kidney, liver, lung, and additional tissue types, along with data generated from the next‐generation Visium HD platform (Figure S3). Visual inspection of marker genes, such as *ACTA2* in kidney and *CDH5* in bone, confirmed that SpotZoomer faithfully recovers continuous tissue structures that are often fragmented by baseline methods (Figure S3b, d, f). To rigorously quantify this performance, we evaluated multiple metrics capturing both perceptual quality and numerical accuracy. SpotZoomer achieved the lowest Mean Absolute Error (MAE) on Visium HD datasets and the highest Structural Similarity Index (SSIM) on Visium datasets (Figure S3g,i). For a comprehensive quantitative assessment, we reported the full distribution of Root Mean Square Error (RMSE) and Pearson Correlation Coefficient (PCC) metrics in Figures S4a and S5a, respectively. These extensive evaluations consistently demonstrate that SpotZoomer minimizes reconstruction errors and maximizes biological fidelity, regardless of the tissue type or sequencing platform employed.

In addition to the Teacher‐prior ablation shown above, we further conducted module‐level ablation studies on the gene encoder (graph contrastive learning), the multimodal fusion module (BMHA), and the cross‐platform alignment module (MNN + triplet loss), each compared against carefully matched alternative‐design baselines. The default SpotZoomer configuration consistently outperformed all variants across four complementary metrics, confirming that every core architectural component contributes a non‐redundant and substantial gain (see Figure S10).

### 2.3 SpotZoomer Enables the Clear Identification of Tumor Boundaries and Tertiary Lymphoid Structures in the Visium Data From Colorectal Cancer

We applied SpotZoomer to enhance the spatial resolution of Visium data from colorectal cancer (CRC). To rigorously evaluate SpotZoomer's super‐resolution capability in a realistic biological setting, rather than relying on pseudo‐downsampling of high‐resolution data. We leveraged a recently published colorectal cancer (CRC) dataset [13] in which two different patient samples were profiled by complementary platforms on adjacent tissue sections. Specifically, Sample 1 was profiled by Visium HD (denoted CRC I in Table S1), and Sample 2 was profiled by both standard Visium (denoted CRC II) and Visium HD on adjacent sections. We used CRC I (Sample 1, Visium HD) as the Teacher domain for learning high‐resolution distributional priors, and CRC II (Sample 2, standard Visium, never overlapping with Sample 1) as the low‐resolution input. Crucially, Sample 2's Visium HD section was held out entirely from training and served exclusively as the empirical ground truth against which SpotZoomer's reconstruction was quantitatively evaluated. This design, where the Teacher prior is learned on one patient and the reconstruction is validated against the adjacent‐section HD profile of a different patient, provides an independent, biologically authentic benchmark of cross‐sample generalization. Quantitative reconstruction metrics on this configuration are reported in Figure S3i (SSIM) and Figure S6a (PCC, RMSE, MAE).

**FIGURE 3:**
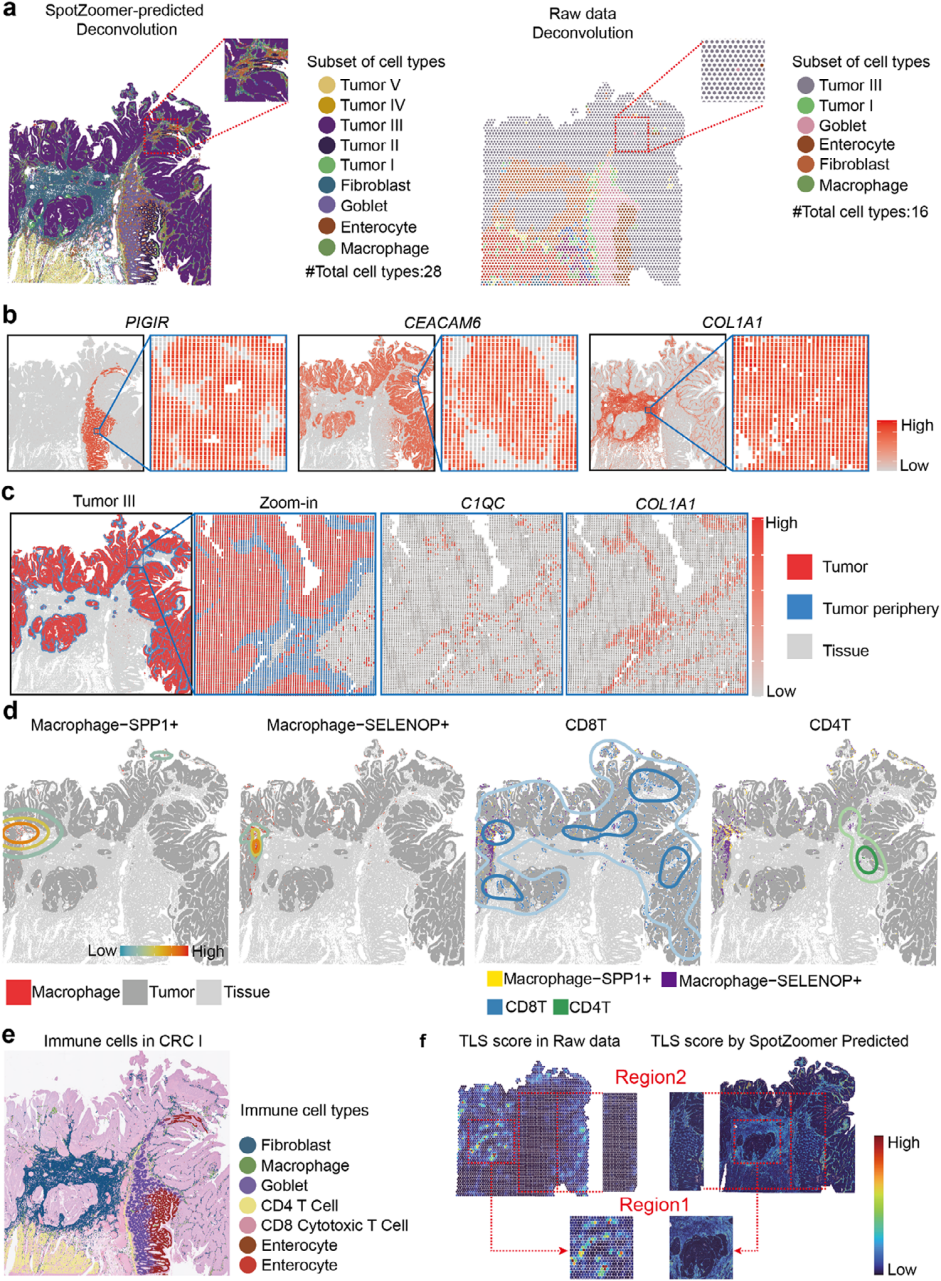
SpotZoomer enhances spatial resolution to resolve fine‐grained cell‐type distributions and immune microenvironment features in tumor tissues. (a) Cell‐type deconvolution from original Visium versus SpotZoomer‐predicted data; higher resolution reveals finer cell‐type composition. (b) Spatial expression of marker genes supporting the deconvolution in (a): PIGR (goblet cells/enterocytes), CEACAM6 (tumor), and COL1A1 (fibroblasts). (c) Tumor boundary analysis. Boundaries (blue) were defined as regions within 50 µm of tumor spots (red), a zone likely containing cells that interact with the tumor or shape the tumor microenvironment (TME). Boundary cellular composition was quantified against the remaining tissue and validated by macrophage (C1QC) and CAF (COL1A1) markers, both significantly upregulated at the boundary (one‐sided Wilcoxon rank‐sum test: C1QC, p = 4.7 × 10^−^^1^ ^8^; COL1A1, p = 1.2 × 10^−^^2^ ^2^). (d) Spatial density of immune cell subsets (SPP1^+^/SELENOP^+^ macrophages, CD8 T, CD4 T), identifying immune‐enriched areas within tumor subregions. (e) Immune‐related cell distribution in CRC II, overlaid at reduced opacity on the matched H&E section to co‐visualize cell distribution with histology (e.g., lymphoid aggregates, tumor margins); a clean H&E inset is shown for reference. (f) TLS detection in CRC I, SpotZoomer‐predicted versus raw data. TLS scores were significantly enriched in SpotZoomer‐identified TLS regions versus non‐TLS regions (one‐sided Wilcoxon rank‐sum test, p = 8.3 × 10^−^^5^ ^7^, r = 0.48); the same test on original Visium data over identical regions yielded a markedly weaker effect (p = 2.1 × 10^−^^9^, r = 0.18), reflecting the limited capacity of native Visium resolution to resolve TLS architecture. TLS marker genes are listed in Table S3. Fields of view: full‐tissue panels in (a–f), ≈ 6.5 × 6.5 mm. Zoom‐in insets: (a) ≈ 500 × 500 µm; (b) ≈ 600 × 600 µm; (c) ≈ 1 × 1 mm (including the C1QC and COL1A1 panels); (f) ≈ 800 × 800 µm.

We compared the spatial data generated by SpotZoomer with the original Visium data to investigate whether the generated data could capture more sophisticated structures. The original Visium data are at 100 µm resolution, while the generated data are at ∼8 µm resolution. We first used a widely used cell type deconvolution method, RCTD [30], to deconvolute these two datasets. We found that major cell types such as fibroblasts, goblet cells, and tumor III cells had consistent spatial patterns (Figure 3a). However, goblet cells were also enriched in the top right corner (circled with a red line) of the spatial data generated by SpotZoomer but not in the original data. The high‐resolution predictions not only substantially improve the delineation of boundaries between tumor and immune cells but also enhance the detection of local heterogeneous structures. For example, in the magnified view, SpotZoomer accurately identifies small immune cell clusters adjacent to the tumor margin, thereby providing a foundation for subsequent microenvironmental analysis.

Subsequently, we assessed the accuracy of the deconvolution outcomes and their concordance with gene‐level expression predicted by SpotZoomer by examining marker gene expression patterns in the spatial transcriptomics data (Figure 3b). We selected three representative marker genes, namely PIGR, CEACAM6, and COL1A1, which represent epithelial secretory function, cancer‐associated epithelial traits, and extracellular matrix remodeling, respectively. In the SpotZoomer‐generated data, the expression of these genes is more spatially concentrated and better aligned with the architectural features of tumor tissue. The magnified view clearly shows that at the tumor–normal interface, *COL1A1* is predominantly localized in the stromal region, whereas *PIGR* and *CEACAM6* are highly enriched within the tumor core, providing a more accurate representation of cell‐type specificity and spatial localization in the SpotZoomer‐generated expression map.

To further demonstrate that the super‐resolution gene expression profiles generated by SpotZoomer offer improved characterization of the tumor edge microenvironment, we defined a tumor boundary region based on the deconvolution results and analyzed the local expression of key genes (Figure 3c). The tumor boundary region was defined as the area within 10 µm of the tumor cell annotation point—a resolution that exceeds the analytical capacity of standard Visium data—focusing on the expression of *C1QC* (immune‐related) and *COL1A1* (stromal‐related). The results show that both *C1QC* and *COL1A1* are significantly upregulated in the boundary region, indicating that this region likely functions as a hub for immune cell infiltration and extracellular matrix remodeling. For example, in tumor zone III, there is a ring‐like enrichment pattern at the tumor edge revealed by SpotZoomer, underscoring the utility of high‐resolution mapping for uncovering local microenvironmental heterogeneity.

We analyzed macrophage‐rich tumor regions to gain insights into the tumor microenvironment (TME) using the generated super‐resolution expression data (Figure 3d). To this end, we selected 10 µm‐sized regions identified as macrophages via deconvolution around the tumor region for independent unsupervised clustering analysis. We identified highly enriched regions using density estimation and observed that SELENOP^+^ and SPP1^+^ macrophages were primarily distributed in distinct spatial regions within the tissue. T cell spot types are challenging to identify at Visium‐level resolution, and their recruitment and functions are closely linked to dynamic changes within the tumor microenvironment [31]. To demonstrate SpotZoomer's ability to resolve spatial distributions of cell types in cancer tissues, we used density maps to display the locations of CD8^+^ and CD4^+^T cells in high‐resolution gene expression profiles.

Finally, we evaluated the ability of SpotZoomer to identify immune‐functional structures, particularly tertiary lymphoid structures (TLS). We focused on the spots enriched with immune cells (Figure 3e). We used a predefined list of TLS marker genes (Table S3) to calculate TLS scores by normalizing and averaging the predicted gene expression levels. We then compared the spatial distribution of the TLS scores in the original Visium data and the SpotZoomer‐generated data (Figure 3f). In Region 1 and Region 2, the original Visium resolution was unable to resolve finer TLS structures. In particular, in Region 2, the high‐resolution gene expression profile predicted by SpotZoomer revealed a more distinct TLS structure. Furthermore, the TLSs predicted and identified by SpotZoomer correspond well with the distribution of immune cell type spots in the tissue. These findings indicate that SpotZoomer not only improves the spatial resolution of immune cell localization but also facilitates the identification of complex immune structures.

### 2.4 SpotZoomer Uncovers the Intricate Cell‐Cell Communication Networks Within Tertiary Lymphoid Structures in Human Breast Cancer

**FIGURE 4:**
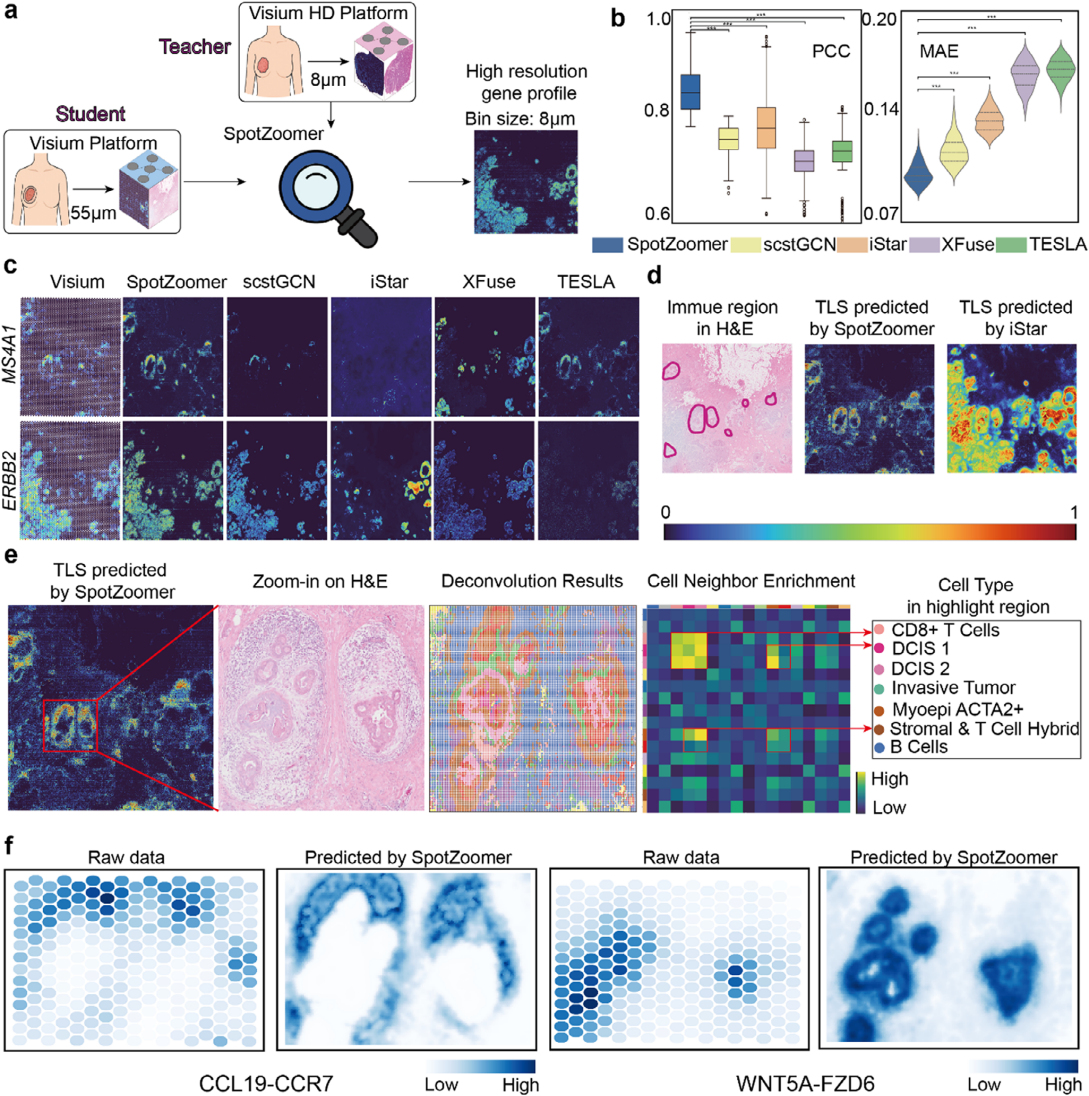
High‐resolution characterization of tertiary lymphoid structures (TLSs) in breast cancer using SpotZoomer. (a) SpotZoomer framework for cross‐platform high‐resolution prediction: Visium data is enhanced to HD‐level resolution (8 ***µ*** m bins) using Visium HD data as reference. (b) Performance evaluation using Pearson Correlation Coefficient (PCC) and Mean Absolute Error (MAE) on Breast II datasets. The box plots show the distribution of predicted high‐resolution gene expression, with the center line indicating the median and the boxes representing the interquartile range (IQR).; SpotZoomer achieves the highest correlation and lowest error. (c) Spatial visualization of TLS marker gene expression patterns (e.g., MS4A1, ERBB2) demonstrates that SpotZoomer outperforms other methods in enhancing fine‐grained spatial detail. (d) TLS scores derived from raw Visium, iStar, and SpotZoomer predictions, showing improved delineation of immune‐rich regions by SpotZoomer. (e) TLS analysis in Zoom‐in region: predicted TLS scores, H&E morphology, cell‐type deconvolution, and neighborhood enrichment analysis reveal cellular organization within TLSs. (f) Comparison of raw data with SpotZoomer‐predicted ligand‐receptor interaction strengths (CCL19‐CCR7 and WNT5A‐FZD6). Physical dimensions of displayed fields of view: (c) gene expression panels (MS4A1, ERBB2 across six methods) correspond to approximately 2.5 × 2.5 mm each. (d) Immune region H&E and TLS prediction panels correspond to approximately 2.5 × 2.5 mm each. (e) Full‐tissue TLS panel corresponds to approximately 6.5 × 6.5 mm; the H&E zoom‐in and deconvolution zoom‐in correspond to approximately 800 × 800 µm each. (f) CCL19–CCR7 and WNT5A–FZD6 raw‐data panels (showing Visium spot grid) correspond to approximately 1.5 × 1.5 mm each; the corresponding SpotZoomer‐predicted panels show the same fields of view.

Next, we applied SpotZoomer to the Breast II dataset, obtained from the Visium platform with a resolution of 55 µm, with guidance from an available Visium HD dataset of breast cancer (Figure 4a). We systematically compared SpotZoomer with XFuse, TESLA, iStar, and scstGCN (Figure 4b). The results show that SpotZoomer outperforms these four methods across multiple evaluation metrics, achieving significantly lower mean absolute error (MAE) and higher Pearson correlation coefficient (PCC), thereby demonstrating its comprehensive superiority in gene expression reconstruction (RMSE and SSIM are reported in Figure S8a). We also visualized two classic human breast cancer marker genes: MS4A1 and ERBB2. Using gene expression data at Visium resolution as input, we reconstructed high‐resolution profiles and found that SpotZoomer preserved spatial expression patterns while enhancing resolution, outperforming other methods (Figure 4c). This improvement stems from the use of a weakly self‐supervised training strategy based on gene expression data, rather than relying solely on image‐derived features to infer high‐resolution expression.

To assess whether the enhanced resolution provided by SpotZoomer enables more accurate delineation of TLS regions, we utilized manually annotated immune cell regions from the original dataset as ground truth (in the H&E panel of Figure 4d) and visualized TLS scores computed by SpotZoomer and iStar (Figure 4d). TLS regions detected in the SpotZoomer‐generated data demonstrated high concordance with ground truth spatial annotations in the annotated immune regions, exhibiting sharp expression patterns and well‐defined spatial boundaries, resulting in the highest identification accuracy among all evaluated methods.

Furthermore, we conducted an analysis of TLS regions detected by SpotZoomer (Figure 4e). In these regions, we constructed cell‐level deconvolution maps based on SpotZoomer‐enhanced gene expression profiles and performed cell‐type enrichment analysis. The results indicated significant immune cell enrichment within TLS regions, thereby functionally validating the identification of TLSs and demonstrating that high‐resolution spatial expression facilitates broader identification of immune cell subtypes, thus overcoming the resolution limitations inherent to the original Visium platform. We then compared cell–cell communication patterns inferred using the original and SpotZoomer‐enhanced data within local TLS regions. Specifically, we visualized the CCL19–CCR7 and WNT5A–FZD6 communication intensities in the raw data and the SpotZoomer‐predicted data (Figure 4f; receptor–ligand interactions are visualized in Figure S9). In the SpotZoomer‐enhanced data, the CCL19–CCR7 signaling intensity was elevated compared to that observed at the original resolution, suggesting that the enhanced regions exhibited advanced TLS maturation, active antigen presentation, robust T‐cell priming, and increased immunotherapeutic potential [32, 33, 34]. This finding was consistent with TLS identification shown in Figure 4d. The WNT5A–FZD6 receptor–ligand pair, in which WNT5A binds to the FZD6 receptor, primarily activates the non‐canonical Wnt signaling pathway, regulating cell migration, cytoskeletal remodeling, polarity, and immune responses, and may exert context‐dependent bidirectional effects in various tumor microenvironments [35, 36, 37]. Both signaling pathways, which were poorly resolved in the original low‐resolution data, exhibited stronger activity and distinct regional distributions in the predicted high‐resolution data. In breast cancer TLSs, CCL19–CCR7 signaling was significantly enhanced in the follicular zone, indicating TLS maturation and heightened immune activation, whereas WNT5A–FZD6 signaling exhibited focal distributions around TLSs or within tumor regions, implying stromal‐mediated immune suppression. Comparison of these two signaling pathways at different resolutions highlights that SpotZoomer‐enhanced high‐resolution expression can identify the dynamic interplay between TLS‐mediated anti‐tumor immunity and tumor stromal suppression.

### 2.5 SpotZoomer Enhances Transcriptomic Resolution and Reveals Stage‐Specific Features of Pulmonary Fibrosis in Different Mouse

**FIGURE 5:**
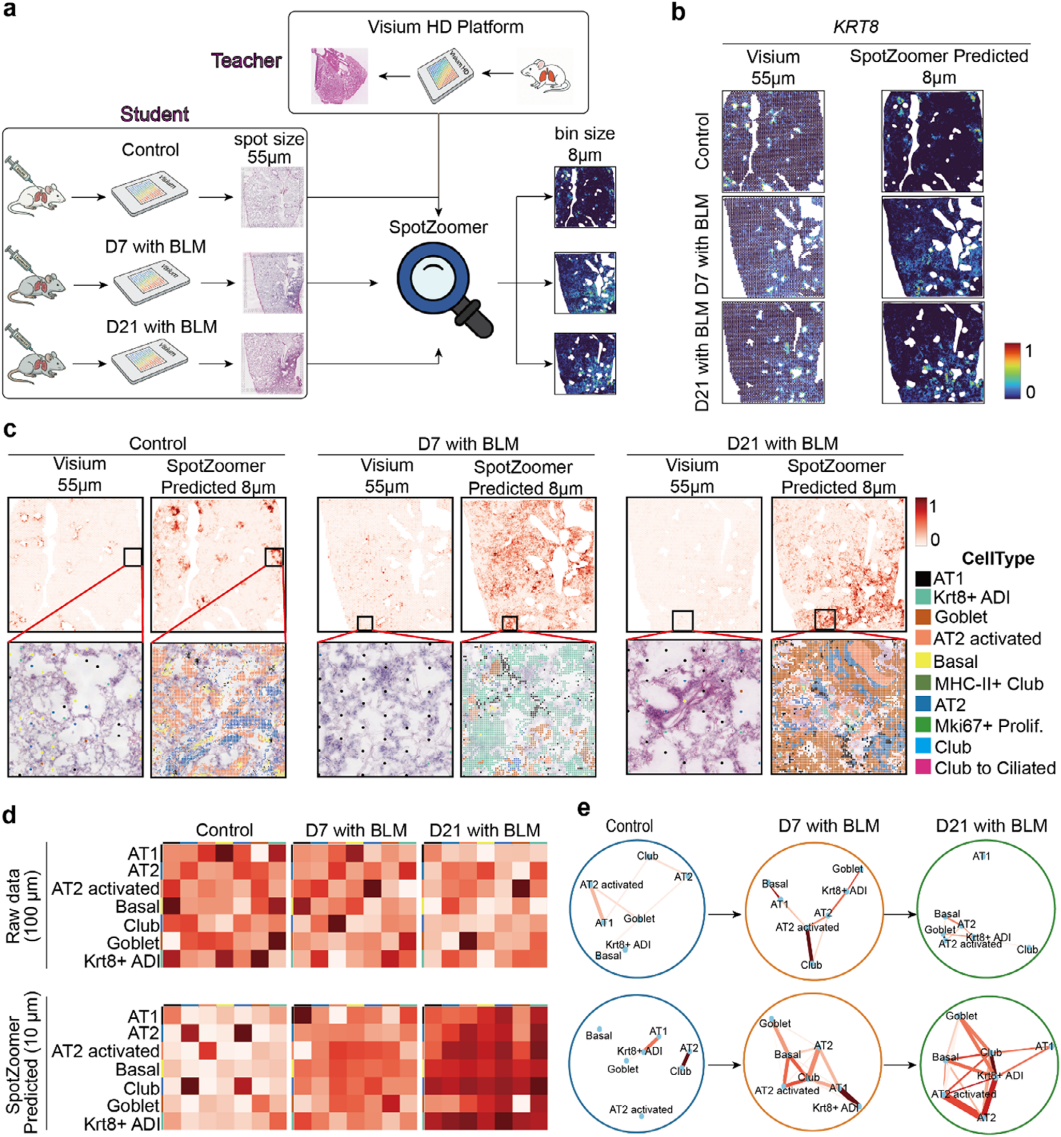
SpotZoomer enhances spatial transcriptome resolution in lungs of different mice across species. (a) Benchmark setup for cross‐species super‐resolution: Visium data is enhanced to HD‐level resolution (8 ***µ*** m bins) using Visium HD data as reference. Control: mice injected with saline, D7 with BLM: mice injected with bleomycin for seven days, D21 with BLM: mice injected with bleomycin for 21 days, Visium HD: lung sections of healthy mice. (b) Spatial visualization of the expression patterns of mouse lung fibrosis marker genes. Compared with the original Visium pattern, SpotZoomer can accurately reconstruct high‐resolution patterns between different samples under the same prior knowledge. (c) Spatial visualization of AT2‐activated cell density and deconvolution results reveals a clearer and more distinct distribution pattern compared to the original Visium data. After resolution enhancement, the cell density becomes more concentrated and structured. Deconvolution analysis further confirms that AT2‐activated cells are highly enriched in the highlighted regions. (d) Heatmaps showing pairwise cell‐type co‐occurrence scores across three experimental conditions: Control, Day 7 with BLM, and Day 21 with BLM. Upper panels represent raw Visium data at 100 ***µ*** m resolution, while lower panels show SpotZoomer‐predicted co‐occurrence at enhanced 10 ***µ*** m resolution. SpotZoomer improves spatial resolution, revealing more distinct and concentrated co‐occurrence patterns, particularly in the Day 21 group. (e) Cell–cell co‐occurrence networks constructed from SpotZoomer‐predicted data for each condition. Nodes represent cell types, and edges denote spatial co‐occurrence relationships, with line thickness corresponding to co‐occurrence strength. Compared to the Control group, BLM‐treated groups exhibit increasingly complex and interconnected networks, indicating progressive spatial reorganization and enhanced interactions among specific cell types, such as AT2 activated, Club, and Krt8+ ADI. Physical dimensions of displayed fields of view: (b) KRT8 expression panels (Visium 55 µm and SpotZoomer‐predicted 8 µm, for Control / D7 with BLM / D21 with BLM) correspond to approximately 6.5 × 6.5 mm each. (c) Full‐tissue cell density panels (Visium and SpotZoomer‐predicted, for all three conditions) correspond to approximately 6.5 × 6.5 mm each; the alveolar‐structure zoom‐in panels correspond to approximately 500 × 500 µm each.

To evaluate the robustness of SpotZoomer in generating super‐resolution data across samples, we applied SpotZoomer to three Visium datasets of mouse lung tissue sections under the guidance of a Visium HD mouse lung section (Lung III) from a healthy mouse. These three Visium datasets come from distinct pathological states and time points, including a healthy control group injected with saline, an early‐stage pulmonary fibrosis group collected 7 days after bleomycin treatment, and a late‐stage fibrosis group collected 21 days post‐treatment (Figure 5a). Using the same reference Visium HD dataset, we visualized the super‐resolution gene expression maps reconstructed by SpotZoomer and compared them with the corresponding Visium‐resolution data (Figure 5b). The results demonstrate that SpotZoomer effectively captures distinct expression patterns across pathological conditions while preserving structural fidelity at high resolution. Given the critical role of AT2‐activated cells in the progression of pulmonary fibrosis [43, 44], we further conducted cell type deconvolution on SpotZoomer‐enhanced spatial gene expression profiles, using single‐cell data as a reference [45]. We visualized the spatial distribution and density of AT2‐activated cells under different pathological conditions (Figure 5c). The differences in spatial morphology and cell density observed across the three samples further demonstrate that SpotZoomer can preserve biologically relevant heterogeneity.

To investigate how intercellular organization evolves with increasing lung injury, we compared the co‐occurrence patterns of different cell types at high resolution (SpotZoomer‐enhanced) versus the original Visium platform under three conditions: control, day 7, and day 21 after BLM treatment (Figure 5d). The co‐occurrence patterns shown in the original Visium data appear diffuse and lack contrast. In contrast, the SpotZoomer‐enhanced map reveals a sharply defined and spatially confined colocalization pattern. The increase in resolution significantly enhances spatial delineation, particularly at day 21 post‐BLM treatment. At this stage, the SpotZoomer‐predicted map indicates increased spatial proximity between AT2‐activated cells and Krt8^+ alveolar differentiation intermediate (ADI) cells, which may suggest a synergistic role in regeneration or pathological remodeling during the later stages of lung injury [38]. These spatial details are difficult to discern in the original low‐resolution data, underscoring the value of high‐resolution approaches in revealing the spatial topology of cellular organization within complex tissue microenvironments. Overall, SpotZoomer not only improves spatial resolution but also facilitates the identification of underlying cellular interaction mechanisms at critical stages of disease progression.

To further analyze the spatial remodeling of lung tissue architecture in response to injury, we constructed cell co‐occurrence networks under different experimental conditions using high‐resolution data predicted by SpotZoomer (Figure 5e). In these networks, nodes represent distinct cell types, and the thickness of the edges indicates the degree of spatial co‐occurrence between cell types. The control group exhibited sparse connectivity and minimal spatial interactions. However, following BLM treatment, particularly at day 21, the network developed into a highly interconnected structure, reflecting substantial remodeling of the tissue microenvironment. Notably, AT2‐activated cells, Club cells, and Krt8^+ alveolar differentiation intermediate (ADI) cells emerged as central hubs within the fibrotic network, demonstrating strong spatial associations with multiple epithelial cell types. This enhanced spatial proximity not only suggests active intercellular communication but also indicates the emergence of synergistic microenvironmental units during injury repair or fibrotic progression. The gradual increase in spatial connectivity from day 7 to day 21 parallels the temporal evolution of lung fibrosis, indicating a transition from sparse cellular distribution to a compact, multicellularly coordinated architecture [39]. These results highlight the critical role of high‐resolution spatial modeling in revealing dynamic cell networks and tissue remodeling that remain largely inaccessible using conventional whole‐tissue analytical approaches [40].

## 3 Discussion

SpotZoomer demonstrates that high‐resolution spatial transcriptomic data can serve not only as a measurement platform, but also as transferable prior knowledge for improving lower‐resolution datasets. By learning from reference high‐definition profiles and adapting this information to standard Visium data, the framework reconstructs spatial expression patterns that are difficult to recover from morphology alone. This distinguishes SpotZoomer from purely reference‐free approaches, while still making it complementary to them: when suitable high‐resolution reference data are available, a reference‐guided strategy can improve biological fidelity; when such references are unavailable, reference‐free prediction remains valuable. Related work using vision‐language contrastive learning to predict expression from H&E images addresses a different task at native Visium resolution [41], whereas SpotZoomer focuses on super‐resolution reconstruction through cross‐platform transfer. We compared with OmiCLIP in Figures S13 and S14.

Across the evaluated datasets, the benefit of SpotZoomer was most evident in biological settings where fine spatial structure is critical. In colorectal cancer, the reconstructed profiles improved the delineation of tumor boundaries, cancer cell subtypes, and local microenvironmental organization. In breast cancer, higher‐resolution predictions enabled clearer characterization of tertiary lymphoid structures and immune‐cell enrichment within tumor regions. In pulmonary fibrosis, SpotZoomer captured disease‐stage‐associated spatial remodeling of AT2‐related expression patterns and provided more detailed co‐occurrence information than native Visium data. These applications suggest that super‐resolution reconstruction can support downstream analyses that depend on spatial precision, including cell‐type mapping, tissue boundary detection, and inference of intercellular communication.

The performance of SpotZoomer also highlights the importance of incorporating molecular constraints into spatial prediction. Histological images provide valuable contextual information, but morphology alone may be insufficient for genes whose expression is sparse, weakly coupled to visible tissue features, or regulated by molecular programs that are not apparent in H&E staining. By combining gene expression, spatial neighborhood structure, image features, and high‐resolution reference priors, SpotZoomer reduces the risk of relying exclusively on visual similarity. This multimodal design helps the model generate reconstructions that are more consistent with observed spatial transcriptomic structure.

Several limitations should be noted. First, SpotZoomer depends on the availability and quality of high‐resolution reference data. Our perturbation analyses showed that the model degrades gradually when cohort size, sequencing depth, capture efficiency, or technical noise is altered, with structural similarity being more robust than value‐level accuracy under stressed conditions. This suggests that SpotZoomer can tolerate moderate imperfections in reference data, but reference quality remains an important determinant of performance. Second, although the current framework integrates transcriptomic, spatial, and histological information, future extensions could incorporate additional modalities such as chromatin accessibility, epigenomic profiles, or spatial proteomics. Third, platforms such as Stereo‐seq and SeekSpace may provide additional teacher domains as more paired high‐resolution and H&E datasets become publicly available, although platform‐specific preprocessing and data harmonization will be necessary.

Overall, SpotZoomer provides a practical strategy for extracting higher‐resolution spatial information from widely available Visium datasets. By bridging standard spot‐level profiling with high‐definition spatial priors, it offers a cost‐effective route to more detailed tissue mapping without requiring every sample to be sequenced using the most expensive high‐resolution platforms. This framework may be especially useful for disease studies where archived or large‐scale Visium cohorts already exist but lack sufficient spatial resolution for cell‐level interpretation. More broadly, SpotZoomer supports a general direction for spatial omics analysis: using expanding public high‐resolution datasets as reusable biological priors to improve the resolution, interpretability, and downstream utility of lower‐resolution spatial data.

## 4 Methods

### 4.1 Data Preprocessing

In this study, we analyzed multiple spatial transcriptomic datasets generated by 10x Genomics, including datasets from the Visium HD, Xenium, and standard Visium platforms (Table S1). Considering the different characteristics of diverse platforms, we developed tailored preprocessing pipelines for each platform and employed the following platform‐adaptive strategies to construct pseudo low‐resolution spatial transcriptomics data.

Visium HD represents the latest high‐resolution spatial transcriptomic technology developed by 10x Genomics. Its sequencing bins are densely arranged square regions without inter‐spot gaps (e.g., 8×8µm), resulting in a higher spatial density compared to the standard Visium platform. Based on the spot diameter of the Visium platform (∼ 55µm) and the spatial interval between adjacent spots (∼ 100µm), we partition the high‐resolution expression data from Visium HD into virtual spots to generate pseudo‐Visium data [42]. Specifically, the gene expression level of a pseudo spot is computed by aggregating the expression levels of all constituent bins within its spatial coverage, as defined by the following formulation:

(1) $$s_{i}=\sum_{\left(x,y\right)}\delta_{i}^{xy}\cdot p_{xy},\delta_{i}^{xy}=\left\{\begin{array}{cc} 1, & \mathrm{if}\;d\left(s_{i},P_{xy}\right)\leq r\\ 0, & \mathrm{else} \end{array}\right.$$

where *p_xy_* denotes the gene expression level at the sequencing unit (pixel) located at coordinates (*x*, *y*). For a given pseudo‐spot *S_i_*, its total gene expression is denoted by *s_i_*. An indicator function $\delta_{i}^{xy}$ equals 1 if pixel *P_xy_* lies within the effective radius of spot *S_i_*, and 0 otherwise. The Euclidean distance between the center of spot *S_i_* and the center of pixel *P_xy_* is represented as *d*(*s_i_*,*P_xy_*). The parameter *r* denotes the physical radius of a spot coming from the Visium platform.

Xenium offers subcellular‐level sequencing resolution while simultaneously providing spatial localization of cellular structures. In the data preprocessing stage, to construct a ground‐truth expression reference, a regular rectangular superpixel grid was constructed. Subsequently, cell‐level gene expression values were proportionally allocated to overlapping grid cells based on the spatial intersection area. The allocation process is defined by the following formula:

(2) $$p_{xy}=\sum_{k}\omega_{xy}^{\left(k\right)}\cdot c_{k},\omega_{xy}^{\left(k\right)}=\frac{A_{xy}^{\left(k\right)}}{A_{k}}$$

where *p_xy_* denotes the gene expression level at the pixel (or super‐pixel) located at position (*x*, *y*). The gene expression profile of the *k*‐th cell is denoted by *c_k_*. The total area of cell *k* is represented by *A_k_*, while $A_{xy}^{\left(k\right)}$ indicates the overlapping area between cell *k* and the pixel at position (*x*, *y*). We define $\omega_{xy}^{\left(k\right)}$ as the normalized overlap weight, representing the proportional contribution of cell *k* to the gene expression observed at location (*x*, *y*). Subsequently, the pseudo‐Visium data of the Xenium platform was generated as:

(3) $$s_{j}=\sum_{k}\eta_{j}^{\left(k\right)}\cdot c_{k},\eta_{j}^{\left(k\right)}=\left\{\begin{array}{cc} 1, & \mathrm{if}\;c_{k}\;\mathrm{is}\;\mathrm{covered}\;\mathrm{by}\;\mathrm{pseudo}\;\mathrm{spot}\;s_{j}\\ 0, & \mathrm{else} \end{array}\right.$$

In this formulation, *s_i_* denotes the gene expression of pseudo‐spot *S_i_*, and *c_k_* represents the gene expression of the *k*‐th cell. The indicator function $\eta_{i}^{\left(k\right)}$ is used to determine whether cell *k* is within the spatial boundary of pseudo‐spot *S_i_*; it takes the value 1 if cell *k* is covered by *S_i_*, and 0 otherwise.

For the Visium platform, which inherently provides spot‐level resolution, we generate pseudo‐subVisium data by down‐sampling the original expression matrix for model input. This procedure mirrors the generation of pseudo‐Visium data from Visium HD, except that the spatial coordinates of each real spot $s_{j}^{\mathrm{true}}$ are directly obtained from the original Visium dataset. Top 3000 highly variable genes were selected in all spot‐based Visium and bin‐based Visium HD dataset, while all available genes were used in Xenium datasets.

### 4.2 The Structure of SpotZoomer

SpotZoomer is a generative model for generating super‐resolution spatial transcriptomics data by transfer learning. It mainly consists of a gene encoder module, an H&E image encoder module, a multimodal feature fusion module, a cross‐platform alignment module, and a high‐resolution gene prediction module.

### 4.3 Gene Encoder Module

To fully exploit the spatial information and gene expression, we adopt an undirected graph *G* = (*V*, *E*) to represent the data, where each node indicates a spot, and edges indicate spatial adjacency relationships $A\in R^{N_{\mathrm{spot}}\times N_{\mathrm{spot}}}$ with a predefined number of neighbors *k*, *N*_spot_ denotes the total number of spots. Inspired by the previous studies [43, 44], we propose a self‐supervised graph contrastive learning framework. Specifically, a Graph Convolutional Network (GCN) is employed as the encoder to capture key features of gene expression patterns and spatial structures. Given the graph *G* and expression matrix *X*, the encoder outputs a latent representation *Z_s_*, which is then reconstructed into an expression matrix $\tilde{X}$ by a symmetric decoder. The propagation rule of the encoder at layer *l* is formulated as:

(4) $$Z_{s}^{l}=\sigma \left(\tilde{A}Z_{s}^{l-1}W_{e}^{l-1}+b_{e}^{l-1}\right),$$

where $\tilde{A}=D^{-\frac{1}{2}}AD^{-\frac{1}{2}}$ is the symmetric normalized adjacency matrix, *D* is the degree matrix, $W_{e}^{l}$ and $b_{e}^{l}$ are trainable weights and biases, respectively, and σ(·) denotes the activation function (e.g., ReLU). The initial input is $Z_{s}^{0}=X$. The latent representation *Z_s_* obtained from the encoder is passed through a decoder for reconstruction, with the decoder's propagation rule as follows:

(5) $${\tilde{X}}_{s}^{t}=\sigma \left(\tilde{A}{\tilde{X}}_{s}^{t-1}W_{d}^{t-1}+b_{d}^{t-1}\right),$$

with ${\tilde{X}}_{s}^{0}=Z_{s}$ and the final reconstruction output $\tilde{X}$. The reconstruction loss is defined as:

(6) $$L_{\mathrm{recon}}=\sum_{i=1}^{N_{\mathrm{spot}}}{∥x_{i}-{\tilde{x}}_{i}∥}_{2}^{2},$$

where *x_i_* and ${\tilde{x}}_{i}$ are the original and reconstructed expression vectors for the *i*‐th spot, respectively.

To enhance the discriminative capacity of learned representations, we introduce a self‐supervised contrastive learning (SCL) module by encouraging sensitivity to local contextual information. We first generate a perturbed graph $\hat{G}=\left(\hat{V},\hat{E}\right)$ by keeping the graph topology *G* unchanged and randomly shuffling the entries of the normalized expression matrix *X* to obtain a perturbed expression matrix $\hat{X}$. Then both the original graph *G* and the perturbed graph *G*′ are independently encoded using a shared GNN encoder to obtain representations $Z_{s},Z_{s^{\prime }}\in R^{N_{\mathrm{spot}}\times d}$. Local context vectors *g_i_* are computed by averaging the embeddings of neighboring nodes, followed by a sigmoid transformation, to form positive pairs (*z_i_*, *g_i_*) and negative pairs (*z*_*i*′_, *g_i_*). The training objective for the discriminator is defined by the binary cross‐entropy (BCE) loss function:

(7) $$L_{\mathrm{SCL}}=-\frac{1}{2N_{\mathrm{spot}}}\sum_{i=1}^{N_{\mathrm{spot}}}\left[\log\Phi \left(z_{i},g_{i}\right)+\log\left(1-\Phi \left(z_{i^{\prime }},g_{i}\right)\right)\right].$$

Considering the consistent topology between *G* and *G*′, a symmetric contrastive loss is further defined to improve robustness:

(8) $$L_{\mathrm{SCL}\_\mathrm{corrupt}}=-\frac{1}{2N_{\mathrm{spot}}}\sum_{i=1}^{N_{\mathrm{spot}}}\left[\log\Phi \left(z_{i^{\prime }},g_{i}\right)+\log\left(1-\Phi \left(z_{i},g_{i}\right)\right)\right].$$

Finally, the overall loss is given by:

(9) $$L_{\mathrm{total}}=L_{\mathrm{recon}}+\lambda \left(L_{\mathrm{SCL}}+L_{\mathrm{SCL}\_\mathrm{corrupt}}\right),$$

where λ is a weight coefficient.

### 4.4 H&E Image Encoder Module

We extract features of histological images using UNI [45], a Vision Transformer‐based pathology foundation model. The choice of UNI was empirically validated against three alternative pathology foundation models (CONCH, MUSK, Virchow) under the same SpotZoomer framework, with UNI consistently achieving the best performance across all four metrics (see Figure S12 and Note S1). Due to variations in spatial resolution across images, each image is first rescaled to a uniform spatial resolution of 0.5 ***µ*** m, such that each 16 × 16 pixel patch approximately corresponds to a single cell. When the scaled image dimensions are not divisible by 224, zero‐padding is applied to ensure compatibility with the model input size of 224. Subsequently, the entire histological image is partitioned into non‐overlapping patches of size 224 × 224 pixels, each patch aligned with a sequencing spot, thereby facilitating alignment between visual features and spatial transcriptomic coordinates. Let ***M*** and ***N*** denote the height and width of the histological image, respectively. The entire image can thus be represented as:

(10) $$Y={\left[Y_{mn}\right]}_{m=1,n=1}^{M/224,N/224},Y\in R^{M\times N\times 3}$$

Each sub‐image (patch) is thus:

(11) $$Y_{mn}\in R^{224\times 224\times 3}$$

After extracting features from all sub‐images, denoted as *Y*′_*mn*_, we perform an inverse flattening operation to obtain the full histological feature map $\tilde{Y}$:

(12)

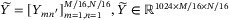




To enhance spatial similarity and robustness among histological images, spatial positional encoding is introduced. We compute positional features based on 2D coordinates of superpixels, forming the positional feature map $P\in R^{2\times M/16\times N/16}$:

(13) $$\mathrm{Pos}\left(k,i,j\right)=\left\{\begin{array}{cc} \frac{\alpha i}{M/16}, & k=0\\ \frac{\alpha j}{N/16}, & k=1 \end{array}\right.$$

where *i* and *j* represent the 2D coordinates of superpixels, and the hyperparameter α is set to 1 empirically.

Finally, by employing average pooling, we down‐sample the entire histological image to the size *M*/16 × *N*/16 to obtain an RGB feature map *R*. We concatenate the histological feature map $\tilde{Y}$, positional feature map *P*, and the RGB feature map *R* along the channel dimension, yielding the comprehensive H&E latent representation:

(14) $$H=\mathrm{concat}\left(\tilde{Y},P,R\right)\in R^{C\times \frac{M}{16}\times \frac{N}{16}}.$$

### 4.5 Multimodal Feature Fusion Module

A Bi‐directional Multi‐Head Attention (BMHA) mechanism [46] is employed to integrate the representations derived from both the gene encoder module and the H&E image encoder module on spatial transcriptomic data. Let $\tilde{X}\in R^{d_{x}\times T}$ and $H\in R^{d_{h}\times T}$ denote the latent representations derived from gene expression and histological features, respectively, where ***T*** is the number of spatial positions (spots), and ***d***_***x***_, ***d***_***h***_ are their respective feature dimensions. We employ standard multi‐head attention to compute cross‐modal attention in both directions:

(15) $$\mathrm{Attentio}n_{1}=\mathrm{MultiHeadAttn}\left(Q=\tilde{X},K=H,V=H\right),$$

(16) $$\mathrm{Attentio}n_{2}=\mathrm{MultiHeadAttn}\left(Q=H,K=\tilde{X},V=\tilde{X}\right),$$

where Attention_1_ represents the attention from gene expression queries to histological features, while Attention_2_ represents the attention from histological queries to gene expression features. This bidirectional design explicitly captures the mutual dependencies between the two modalities. To further enhance the semantic alignment and contextual awareness of the fused representation, the two attention outputs are concatenated and passed through an additional self‐attention layer to produce the final fused representation *X*_f_ as following:

(17) $$X_{f}=\mathrm{SelfAttn}\left(\mathrm{concat}\left(\mathrm{Attentio}n_{1},\mathrm{Attentio}n_{2}\right)\right).$$

The fused representation *X*_f_ captures complementary information from both modalities in a unified feature space. This fusion strategy is identically applied to both pseudo low‐resolution and low‐resolution spatial data, yielding $X_{f}^{src}$ and $X_{f}^{tgt}$, representing the fused representations of the pseudo low‐resolution and low‐resolution spatial data, respectively.

### 4.6 Cross‐Platform Alignment Module

To align fused representations from the low‐resolution data $X_{f}^{\mathrm{tgt}}$ with those from the pseudo low‐resolution data $X_{f}^{\mathrm{src}}$, we design a representation alignment strategy inspired by prior studies [47, 48, 49, 50]. Specially, we apply a MNN strategy to identify anchor‐positive pairs. Let $x_{i}^{\mathrm{src}}\in X_{f}^{\mathrm{src}}$ and $x_{j}^{\mathrm{tgt}}\in X_{f}^{\mathrm{tgt}}$ denote fused feature vectors from the pseudo low‐resolution data and low‐resolution data, respectively. We compute the cosine distance between the fused representations and select MNN pairs based on the following bidirectional mutual‐neighbor criterion:

(18) $$x_{j}^{tgt}\in NN_{k}\left(x_{i}^{src}\right)\mathrm{and}x_{i}^{src}\in NN_{k}\left(x_{j}^{tgt}\right),$$

where NN_*k*_(·) denotes the top‐*k* nearest neighbors under cosine distance. For each anchor $x_{i}^{src}$, we select a negative sample $x_{k}^{tgt}\in X_{f}^{tgt}$ which is not a positive match but exhibits high similarity to the anchor, following a hard negative mining strategy. The alignment objective is formulated as the following triplet loss function:

(19) $$L_{triplet}=\sum_{\left(i,j,k\right)}\max\left(0,\cos\left(x_{i}^{src},x_{k}^{tgt}\right)-\cos\left(x_{i}^{src},x_{j}^{tgt}\right)+\delta \right),$$

where cos(*a*, *b*) denotes the cosine similarity between vectors *a* and *b*, and δ is a margin hyperparameter enforcing a minimum separation between positive and negative pairs. By minimizing $L_{triplet}$, the fused representations of the pseudo low‐resolution data and low‐resolution data are encouraged to align together.

### 4.7 High‐Resolution Gene Prediction Module

This module takes the fused representation of the pseudo low‐resolution data $X_{f}^{\mathrm{src}}$ as input and reconstructs the high‐resolution data ***X***_sr_. The module is realized as a feedforward neural network incorporating residual connections. The computation is formulated as:

(20) $$X_{\mathrm{sr}}=SRPredictor\left(X_{f}^{src}\right)=W_{2}\;\sigma \left(W_{1}X_{f}^{src}+b_{1}\right)+X_{f}^{src},$$

where $W_{1}\in R^{d_{h}\times d_{mid}}$ and $W_{2}\in R^{d_{mid}\times d_{g}}$ are the weight matrices of a two‐layer feedforward network, *b*_1_ is a bias term, and σ(·) denotes a non‐linear activation function ReLU. Here, *d_h_* is the dimensionality of the fused features, *d_mid_* is the hidden layer size, and *d_g_* is the dimensionality of the predicted gene expression. The residual connection preserves spatial structural information from the original fused features and enables the network to capture complex gene expression patterns through non‐linear transformations. The high‐resolution gene prediction module is pre‐trained on pseudo low‐resolution data via supervised learning. Specifically, the fused representation $X_{f}^{src}$ is inputted to the module, and the corresponding gene expression values from the high‐resolution platforms (Visium HD, Xenium) serve as ground‐truth. Let *X*_sr_ denote the predicted super‐resolution gene expression and *X*_gt_ represent the corresponding ground‐truth expression profile obtained from Visium HD or Xenium. The training objective is defined using the mean squared error (MSE), also known as the L2 loss. The loss function is given by:

(21) $$L_{\mathrm{src}}=\frac{1}{N}\sum_{i=1}^{N}∥X_{\mathrm{sr}}^{\left(i\right)}-X_{\mathrm{gt}}^{\left(i\right)}{∥}_{2}^{2},$$

where *N* is the number of spots.

After pre‐trained the gene predicted module on the high‐resolution platform, we finetune the final layer of the module on the low‐resolution platform with a weakly self‐supervised learning strategy. Specially, let *S* denote the total number of spots in the image and *K* the number of genes to be predicted. The expression prediction function for the *k*‐th gene is denoted as *g_k_*, and *y_ks_* represents the observed expression of gene *k* at spot *s*. $M_{s}$ denotes the set of superpixels covered by spot *s*, and *h_nm_* is the fused representation at location (*m*, *n*). The predicted expression of gene *k* at spot *s* is given by:

(22) $${\hat{y}}_{ks}=\sum_{\left(m,n\right)\in M_{s}}g_{k}\left(h_{nm}\right).$$

The final layer of the model is finetuned by minimizing the L2 distance Between predicted and observed gene expression values:

(23) $$L_{\mathrm{wsl}}=\frac{1}{S\times K}\sum_{s=1}^{S}\sum_{k=1}^{K}{\left({\hat{y}}_{ks}-y_{ks}\right)}^{2}$$

Unlike the fully supervised training used on high‐resolution platform, this stage adopts a multi‐task joint optimization strategy to improve generalization and training stability. The overall loss function consists of three components: the triplet loss $L_{\mathrm{triplet}}$, which enforces structural consistency between the fused representations of the low‐resolution and high‐resolution platforms; the reconstruction loss $L_{\mathrm{rec}}$, which reconstructs the fusion representation of low‐resolution platforms; and the weakly self‐supervised loss $L_{\mathrm{wsl}}$, which supervises the prediction of spot‐level gene expression. The overall training objective in the low‐resolution platforms defined as:

(24) $$L_{\mathrm{tgt}}=\lambda_{1}L_{\mathrm{triplet}}+\lambda_{2}L_{\mathrm{rec}}+\lambda_{3}L_{\mathrm{wsl}}$$

where λ_1_, λ_2_, and λ_3_ are hyperparameters that balance the contributions of each loss component.

### 4.8 Training Strategy in SpotZoomer

All SpotZoomer experiments reported in this manuscript were trained with the AdamW optimizer (β_1_ = 0.9, β_2_ = 0.999, ε = 1e‐8) at an initial learning rate of 1e‐3, with a cosine‐annealing learning‐rate schedule decaying to 1e‐5 over the full training horizon. We used a weight decay of 1e‐4 and gradient clipping at a global L2 norm of 1.0 to stabilize training. Training used mini‐batch stochastic optimization with a batch size of **1024** spots randomly sampled from the current slice at each gradient step (drawn without replacement within each epoch). The total number of training epochs E was set to 200 for all reported experiments; for very large slices (≥10,000 spots) E was reduced to 150 to maintain comparable wall‐clock training time without compromising convergence (verified by monitoring the validation loss plateau). All other hyperparameters were held constant across all datasets reported in this manuscript. The full hyperparameter listing is provided in Table S5.

### 4.9 Computational Efficiency

SpotZoomer was trained on a Linux server (Ubuntu 22.04.3 LTS) equipped with an Intel Xeon Platinum 8180 CPU @ 2.50 GHz and an NVIDIA A100 80GB PCIe GPU (Driver Version: 570.148.08, CUDA Version: 12.8). Although SpotZoomer exhibits slightly lower computational efficiency than iStar and scstGCN, it is approximately 300 times faster than TESLA and XFuse.

Across all 19 evaluated datasets, SpotZoomer requires on average ∼5 min of end‐to‐end runtime and ∼3 GB of GPU memory per dataset, well within the capacity of a standard consumer‐grade GPU substantially faster than image‐only baselines such as iStar (∼13 h) and XFuse (∼25 h) while preserving the gene‐encoder advantages from the Teacher prior (see Table S4 for the full comparison). Detailed results on runtime and memory usage are provided in Table S4.

### 4.10 Evaluation Metrics

#### 4.10.1 Recovery Accuracy Evaluation with Root Mean Square Error (RMSE)

RMSE is a commonly used metric to evaluate the difference between predicted and true values by computing the square root of the average squared differences. Compared to MSE, RMSE preserves the original unit of measurement, making it more interpretable. An RMSE close to 0 indicates a high‐fidelity reconstruction. In the implementation, we applied min‐max normalization to the input vectors and used the mean_squared_error function followed by square rooting from the sklearn.metrics Python package (v1.2.0).

#### 4.10.2 Recovery Accuracy Evaluation with Mean Absolute Error (MAE)

MAE calculates the average absolute difference between predicted and true values. It reflects the magnitude of reconstruction errors regardless of direction and is less sensitive to outliers than RMSE. A lower MAE indicates better reconstruction accuracy. In the implementation, we used the mean_absolute_error function from the sklearn.metrics Python package (v1.2.0).

#### 4.10.3 Recovery Accuracy Evaluation with Pearson Correlation Coefficient (PCC)

Pearson correlation coefficient is a metric to quantify the consistency between two variables. A coefficient close to 1 indicates a strong positive linear correlation of spatial patterns between reconstructed results and ground truth. In the implementation, we used the pearsonr function from the scipy.stats Python package (v1.10.0).

Recovery accuracy evaluation with structural similarity index measure (SSIM). SSIM is designed to measure the perceptual similarity between two images, taking into account luminance, contrast, and structural information. It is especially useful for evaluating spatial gene expression maps as images. SSIM values range from –1 to 1, with higher values indicating greater similarity. In the implementation, we used the structural_similarity function from the skimage.metrics Python package (v0.19.3).

### 4.11 Comparative Methods

#### 4.11.1 iStar

iStar is a deep regression model designed to enhance the spatial resolution of transcriptomic data by minimizing the mean squared errors (MSEs) between the aggregated predicted gene expression within each spot and the observed spot‐level gene expression. In our experiments, we utilized the publicly available Python implementation of iStar (https://github.com/daviddaiweizhang/istar) and executed the model using its default configuration settings.

#### 4.11.2 XFuse

XFuse is a deep generative framework that jointly models histological image features and spatial transcriptomic signals to infer high‐resolution gene expression maps. It leverages a shared latent space to enable coherent reconstruction across modalities. In our study, we adopted the official Python implementation (v0.2.1) and performed fine‐tuning using the default hyperparameter settings (https://github.com/ludvb/xfuse).

#### 4.11.3 TESLA

TESLA is a deep learning model developed to improve the spatial resolution of transcriptomic data by learning the underlying gene expression patterns at finer granularity. In our experiments, we used the official Python implementation of TESLA (https://github.com/jianhuupenn/TESLA) and trained the model using its default configuration settings provided by the authors.

#### 4.11.4 scstGCN

scstGCN is a graph‐based deep learning model that improves the spatial resolution of transcriptomic data. In our experiments, we employed the official Python implementation of scstGCN (https://github.com/wenwenmin/scstGCN) and trained the model using the default parameter configuration provided by the authors.

### 4.12 Other Software Packages Used in This Study

Visualization of gene expression at single cell resolution using Cellscopes (https://github.com/HaojiaWu/CellScopes.jl); Deconvolution on high resolution data using RCTD (https://github.com/dmcable/spacexr); Tumor and macrophage subtype analysis using Seurat (https://satijalab.org/seurat); Cell neighborhood enrichment analysis and differential analysis using Squipdy (https://squidpy.readthedocs.io/en/stable/); Cell communication analysis using Regchat (https://github.com/lhzhanglabtools/RegChat).

## Author Contributions

**Lihua Zhang**: conceptualization, methodology, funding acquisition, project administration, writing – review and editing. **Wenwen Min**: conceptualization, methodology, funding acquisition, project administration, writing – review and editing. **Xiaoyu Li**: methodology, software, validation, formal analysis, visualization, writing – original draft.

## Supporting information




**Supporting File**: advs76601‐sup‐0001‐SuppMat.pdf.

## Data Availability

The data that support the findings of this study are available on request from the corresponding author. The data are not publicly available due to privacy or ethical restrictions.

## References

[1] A. Rao , D. Barkley , G. S. França , and I. Yanai , “Exploring Tissue Architecture Using Spatial Transcriptomics,” Nature 596, no. 7871 (2021): 211–220, 10.1038/s41586-021-03634-9. 34381231 PMC8475179

[2] D. J. Burgess , “Spatial Transcriptomics Coming of Age,” Nature Reviews Genetics 20, no. 6 (2019): 317–317, 10.1038/s41576-019-0129-z. 30980030

[3] N. Crosetto , M. Bienko , and A. Van Oudenaarden , “Spatially Resolved Transcriptomics and Beyond,” Nature Reviews Genetics 16, no. 1 (2015): 57–66, 10.1038/nrg3832. 25446315

[4] J. Faupel‐Badger , I. Kohaar , M. Bahl , et al., “Defining Precancer: a Grand Challenge for the Cancer Community,” Nature Reviews Cancer 24, no. 11 (2024): 792–809, 10.1038/s41568-024-00744-0. 39354069

[5] Z. Cang , Y. Zhao , A. A. Almet , et al., “Screening Cell–Cell Communication in Spatial Transcriptomics via Collective Optimal Transport,” Nature Methods 20, no. 2 (2023): 218–228, 10.1038/s41592-022-01728-4. 36690742 PMC9911355

[6] R. Moncada , D. Barkley , F. Wagner , et al., “Integrating Microarray‐Based Spatial Transcriptomics and Single‐Cell RNA‐seq Reveals Tissue Architecture in Pancreatic Ductal Adenocarcinomas,” Nature Biotechnology 38, no. 3 (2020): 333–342, 10.1038/s41587-019-0392-8. 31932730

[7] A. Chen , S. Liao , M. Cheng , et al., “Spatiotemporal Transcriptomic Atlas of Mouse Organogenesis Using DNA Nanoball‐Patterned Arrays,” Cell 185, no. 10 (2022): 1777–1792.e21, 10.1016/j.cell.2022.04.003. 35512705

[8] X. Wang , W. E. Allen , M. A. Wright , et al., “Three‐Dimensional Intact‐Tissue Sequencing of Single‐Cell Transcriptional States,” Science 361, no. 6400 (2018): aat5691, 10.1126/science.aat5691. PMC6339868 29930089

[9] J. R. Moffitt , D. Bambah‐Mukku , S. W. Eichhorn , et al., “Molecular, Spatial, and Functional Single‐Cell Profiling of the Hypothalamic PREOPTIC region,” Science 362, no. 6416 (2018): aau5324, 10.1126/science.aau5324. PMC6482113 30385464

[10] Y. Dong , C. Saglietti , Q. Bayard , et al., “Transcriptome Analysis of Archived Tumors by Visium, GeoMx DSP, and Chromium Reveals Patient Heterogeneity,” Nature Communications 16, no. 1 (2025): 4400, 10.1038/s41467-025-59005-9. PMC12069714 40355415

[11] K. R. Maynard , L. Collado‐Torres , L. M. Weber , et al., “Transcriptome‐Scale Spatial Gene Expression in the Human Dorsolateral Prefrontal Cortex,” Nature Neuroscience 24, no. 3 (2021): 425–436, 10.1038/s41593-020-00787-0. 33558695 PMC8095368

[12] S. Marco Salas , L. B. Kuemmerle , C. Mattsson‐Langseth , et al., “Optimizing Xenium In Situ Data Utility by Quality Assessment and Best‐Practice Analysis Workflows,” Nature Methods 22, no. 4 (2025): 813–823, 10.1038/s41592-025-02617-2. 40082609 PMC11978515

[13] M. F. D. Oliveira , J. P. Romero , M. Chung , et al., “High‐Definition Spatial Transcriptomic Profiling of Immune Cell Populations in Colorectal Cancer,” Nature Genetics 57, no. 6 (2025): 1512–1523, 10.1038/s41588-025-02193-3. 40473992 PMC12165841

[14] L. Tian , F. Chen , and E. Z. Macosko , “The Expanding Vistas of Spatial Transcriptomics,” Nature Biotechnology 41, no. 6 (2023): 773–782, 10.1038/s41587-022-01448-2. PMC10091579 36192637

[15] C. G. Williams , H. J. Lee , T. Asatsuma , R. Vento‐Tormo , and A. Haque , “An Introduction to Spatial Transcriptomics for Biomedical Research,” Genome Medicine 14, no. 1 (2022): 68, 10.1186/s13073-022-01075-1. 35761361 PMC9238181

[16] S. Nagasawa , J. Zenkoh , Y. Suzuki , and A. Suzuki , “Spatial Omics Technologies for Understanding Molecular Status Associated With Cancer Progression,” Cancer Science 115, no. 10 (2024): 3208–3217, 10.1111/cas.16283. 39042942 PMC11447966

[17] C.‐L. Guo , C.‐S. Wang , Z.‐C. Wang , et al., “Granzyme K+CD8+ T Cells Interact With Fibroblasts to Promote Neutrophilic Inflammation in Nasal Polyps,” Nature Communications 15, no. 1 (2024): 10413, 10.1038/s41467-024-54685-1. PMC11607458 39614076

[18] J. T. Ash , G. Darnell , D. Munro , and B. E. Engelhardt , “Joint Analysis of Expression Levels and Histological Images Identifies Genes Associated With Tissue Morphology,” Nature Communications 12, no. 1 (2021): 1609, 10.1038/s41467-021-21727-x. PMC7952575 33707455

[19] B. Schmauch , A. Romagnoni , E. Pronier , et al., “A deep Learning Model to Predict RNA‐Seq Expression of Tumours From Whole Slide Images,” Nature Communications 11, no. 1 (2020): 3877, 10.1038/s41467-020-17678-4. PMC7400514 32747659

[20] L. Bergenstråhle , B. He , J. Bergenstråhle , et al., “Super‐Resolved Spatial Transcriptomics by Deep Data Fusion,” Nature Biotechnology 40, no. 4 (2022): 476–479, 10.1038/s41587-021-01075-3. 34845373

[21] J. Hu , K. Coleman , D. Zhang , et al., “Deciphering Tumor Ecosystems at Super Resolution From Spatial Transcriptomics With TESLA,” Cell Systems 14, no. 5 (2023): 404–417, 10.1016/j.cels.2023.03.008. 37164011 PMC10246692

[22] D. Zhang , A. Schroeder , H. Yan , et al., “Inferring Super‐Resolution Tissue Architecture by Integrating Spatial Transcriptomics With Histology,” Nature Biotechnology 42, no. 9 (2024): 1372–1377, 10.1038/s41587-023-02019-9. PMC11260191 38168986

[23] S. Xue , F. Zhu , J. Chen , and W. Min , “Inferring Single‐Cell Resolution Spatial Gene Expression via Fusing Spot‐Based Spatial Transcriptomics, Location, and Histology Using GCN,” Briefings in Bioinformatics 26, no. 1 (2024): bbae630, 10.1093/bib/bbae630. 39656774 PMC11645551

[24] Y. Wang , B. Liu , G. Zhao , et al., “Spatial Transcriptomics: Technologies, Applications and Experimental Considerations,” Genomics 115, no. 5 (2023): 110671, 10.1016/j.ygeno.2023.110671. 37353093 PMC10571167

[25] W.‐T. Chen , A. Lu , K. Craessaerts , et al., “Spatial Transcriptomics and In Situ Sequencing to Study Alzheimer's Disease,” Cell 182, no. 4 (2020): 976–991, 10.1016/j.cell.2020.06.038. 32702314

[26] S. Jain and M. T. Eadon , “Spatial Transcriptomics in Health and Disease,” Nature Reviews Nephrology 20, no. 10 (2024): 659–671, 10.1038/s41581-024-00841-1. 38719971 PMC11392631

[27] X. Yu , X. Xu , J. Zhang , and X. Li , “Batch Alignment of Single‐Cell Transcriptomics Data Using Deep Metric Learning,” Nature Communications 14, no. 1 (2023): 960, 10.1038/s41467-023-36635-5. PMC9944958 36810607

[28] X. Wang , Y. Chen , and W. Zhu , “A Survey on Curriculum Learning,” IEEE Transactions on Pattern Analysis and Machine Intelligence 44, no. 9 (2022): 1–1, 10.1109/TPAMI.2021.3069908. 33788677

[29] Y. Zhang and Q. Yang , “An Overview of Multi‐Task Learning,” National Science Review 5, no. 1 (2018): 30–43, 10.1093/nsr/nwx105.

[30] D. M. Cable , E. Murray , L. S. Zou , et al., “Robust Decomposition of Cell Type Mixtures in Spatial Transcriptomics,” Nature Biotechnology 40, no. 4 (2022): 517–526, 10.1038/s41587-021-00830-w. PMC8606190 33603203

[31] S.‐X. Ma , L. Li , H. Cai , T.‐K. Guo , and L.‐S. Zhang , “Therapeutic Challenge for Immunotherapy Targeting Cold Colorectal Cancer: A Narrative Review,” World Journal of Clinical Oncology 14, no. 2 (2023): 81–88, 10.5306/wjco.v14.i2.81. 36908678 PMC9993140

[32] Y. Zhang , G. Liu , Q. Zeng , et al., “CCL19‐Producing Fibroblasts Promote Tertiary Lymphoid Structure Formation Enhancing Anti‐Tumor IgG Response in Colorectal Cancer Liver Metastasis,” Cancer Cell 42, no. 8 (2024): 1370–1385, 10.1016/j.ccell.2024.07.006. 39137726

[33] S.‐Y. Wu , S.‐W. Zhang , D. Ma , et al., “CCL19+ Dendritic Cells Potentiate Clinical Benefit of Anti‐PD‐(L)1 Immunotherapy in Triple‐Negative Breast Cancer,” Med 4, no. 6 (2023): 373–393, 10.1016/j.medj.2023.04.008. 37201522

[34] C. Y. C. Lee , B. C. Kennedy , N. Richoz , et al., “Tumour‐Retained Activated CCR7+ Dendritic Cells are Heterogeneous and Regulate Local Anti‐Tumour Cytolytic Activity,” Nature Communications 15, no. 1 (2024): 682, 10.1038/s41467-024-44787-1. PMC10808534 38267413

[35] Y. Chen , Z. Chen , Y. Tang , and Q. Xiao , “The Involvement of Noncanonical Wnt Signaling in Cancers,” Biomedicine & Pharmacotherapy 133 (2021): 110946, 10.1016/j.biopha.2020.110946. 33212376

[36] P. Lopez‐Bergami and G. Barbero , “The Emerging role of Wnt5a in the Promotion of a Pro‐Inflammatory and Immunosuppressive Tumor Microenvironment,” Cancer and Metastasis Reviews 39, no. 3 (2020): 933–952, 10.1007/s10555-020-09878-7. 32435939

[37] G. Corda , G. Sala , R. Lattanzio , et al., “Functional and Prognostic Significance of the Genomic Amplification of Frizzled 6 (FZD6) in Breast Cancer,” The Journal of Pathology 241, no. 3 (2017): 350–361, 10.1002/path.4841. 27859262 PMC5248601

[38] J. G. Wagner , R. P. Lewandowski , and J. R. Harkema , “Anti‐IL5 Antibody Depletes Eosinophils, Promotes Alveolar Type 2 Cell Proliferation, and Attenuates Krt8+ Transitional Epithelial Cells and Fibrosis in Ozone‐Exposed Diabetic Mice,” American Journal of Respiratory and Critical Care Medicine 211, no. 1 (2025): A5090–A5090, 10.1164/ajrccm.2025.211.abstracts.a5090.

[39] Y. Tian , J. Lv , Z. Su , et al., “LRRK2 Plays Essential Roles in Maintaining Lung Homeostasis and Preventing the Development of Pulmonary Fibrosis,” Proceedings of the National Academy of Sciences 118, no. 35 (2021): 2106685118, 10.1073/pnas.2106685118. PMC8536316 34446559

[40] Y. Enomoto , H. Katsura , T. Fujimura , et al., “Autocrine TGF‐β‐Positive Feedback in Profibrotic AT2‐Lineage Cells plays a Crucial Role in Non‐Inflammatory Lung Fibrogenesis,” Nature Communications 14, no. 1 (2023): 4956, 10.1038/s41467-023-40617-y. PMC10471635 37653024

[41] W. Chen , P. Zhang , T. N. Tran , et al., “A Visual–Omics Foundation Model to Bridge Histopathology with Spatial Transcriptomics,” Nature Methods 22, no. 7 (2025): 1568–1582, 10.1038/s41592-025-02707-1. 40442373 PMC12240810

[42] K. Polanski , R. Bartolomé‐Casado , I. Sarropoulos , et al., “Bin2cell Reconstructs Cells From High Resolution Visium HD Data,” Bioinformatics 40, no. 9 (2024): btae546, 10.1093/bioinformatics/btae546. 39250728 PMC11419951

[43] Y. Long , K. S. Ang , M. Li , et al., “Spatially Informed Clustering, Integration, and Deconvolution of Spatial Transcriptomics With GraphST,” Nature Communications 14, no. 1 (2023): 1155, 10.1038/s41467-023-36796-3. PMC9977836 36859400

[44] Q. Zhang , C. Huang , L. Xia , Z. Wang , S. Yiu , and R. Han , “Spatial‐Temporal Graph Learning With Adversarial Contrastive Adaptation,” in Proceedings of the 40th International Conference on Machine Learning, Proceedings of Machine Learning Research (2023), 41151–41163, https://proceedings.mlr.press/v202/zhang23p.html.

[45] R. J. Chen , T. Ding , M. Y. Lu , et al., “Towards a General‐Purpose Foundation Model for Computational Pathology,” Nature Medicine 30, no. 3 (2024): 850–862, 10.1038/s41591-024-02857-3. PMC11403354 38504018

[46] W. Min , Z. Shi , J. Zhang , J. Wan , and C. Wang , “Multimodal Contrastive Learning for Spatial Gene Expression Prediction Using Histology Images,” Briefings in Bioinformatics 25, no. 6 (2024): bbae551, 10.1093/bib/bbae551. 39471412 PMC11952928

[47] L. M. Simon , Y.‐Y. Wang , and Z. Zhao , “Integration of Millions of Transcriptomes Using Batch‐Aware Triplet Neural Networks,” Nature Machine Intelligence 3, no. 8 (2021): 705–715, 10.1038/s42256-021-00361-8.

[48] X. Zhou , K. Dong , and S. Zhang , “Integrating Spatial Transcriptomics Data Across Different Conditions, Technologies and Developmental Stages,” Nature Computational Science 3, no. 10 (2023): 894–906, 10.1038/s43588-023-00528-w. 38177758

[49] G. Kang , L. Jiang , Y. Yang , and A. G. Hauptmann , “Contrastive Adaptation Network for Unsupervised Domain Adaptation,” in Proceedings of the IEEE/CVF Conference on Computer Vision and Pattern Recognition (2019), 4893–4902, https://openaccess.thecvf.com/content_CVPR_2019/html/Kang_Contrastive_Adaptation_Network_for_Unsupervised_Domain_Adaptation_CVPR_2019_paper.html.

[50] Y. Cheng , Y. Su , Z. Yu , Y. Liang , K.‐C. Wong , and X. Li , “Unsupervised Deep Embedded Fusion Representation of Single‐Cell Transcriptomics,” in Proceedings of the AAAI Conference on Artificial Intelligence (2023), 5036–5044, 10.1609/aaai.v37i4.25631.

