# Supplementary material for: Enhancing Super‐Resolution Spatial Transcriptomics Data by Transfer Learning
Source: Adv Sci (Weinh). 2026 Jul 20:e76601. Online ahead of print. doi: 10.1002/advs.76601 (PMC13383706; doi:10.1002/advs.76601)
Supplement: Supplementary file 1 — Supporting File: advs76601‐sup‐0001‐SuppMat.pdf. [file ADVS-9999-e76601-s001.pdf]

Supplementary Materials

Supplementary Figure 1. SpotZoomer structure.

Supplementary Figure 2. Benchmark Setup.

Supplementary Figure 3: Evaluation of SpotZoomer on spatial transcriptomics datasets from multiple tissues and platforms.

Supplementary Figure 4. Evaluation of SpotZoomer on multiple spatial transcriptomics datasets from Xenium platform.

Supplementary Figure 5. Evaluation of SpotZoomer on multiple spatial transcriptomics datasets from Visium HD platform and marker gene visualization.

Supplementary Figure 6. Evaluation of SpotZoomer on multiple spatial transcriptomics datasets from Visium platform and marker gene visualization.

Supplementary Figure 7. Visualization of single-cell level gene expression prediction and evaluation of prediction accuracy.

Supplementary Figure 8. Super-resolution gene expression prediction accuracy on Breast II by different methods and its cell type annotation.

Supplementary Figure 9*.* Visual comparison of the communication intensities of the WNT5A–FZD6 and CCL19–CCR7 receptor-ligand pairs in raw data and high-resolution SpotZoomer predictions.

Supplementary Figure 10. Module-level ablation studies of SpotZoomer's core architectural components.

Supplementary Figure 11. Sensitivity of SpotZoomer to reference (Teacher) data quality and quantity.

Supplementary Figure 12. Ablation of the H&E vision encoder.

Supplementary Figure 13. Baseline comparison at Visium-spot resolution, OmiCLIP's native operating regime.

Supplementary Figure 14. Baseline comparison at high-resolution Visium HD / Xenium level, the super-resolution regime.

Supplementary Figure 15. Cross-species transferability of SpotZoomer.

Supplementary Table 1. Dataset for SpotZoomer training and evaluation.

Supplementary Table 2. Analyze the dataset used in the experiment.

Supplementary Table 3. TLS marker genes.

Supplementary Table 4. Runtime and memory consuming of SpotZoomer on different sequencing platforms.

Supplementary Table 5. Complete hyperparameter settings for all SpotZoomer experiments reported in this manuscript.

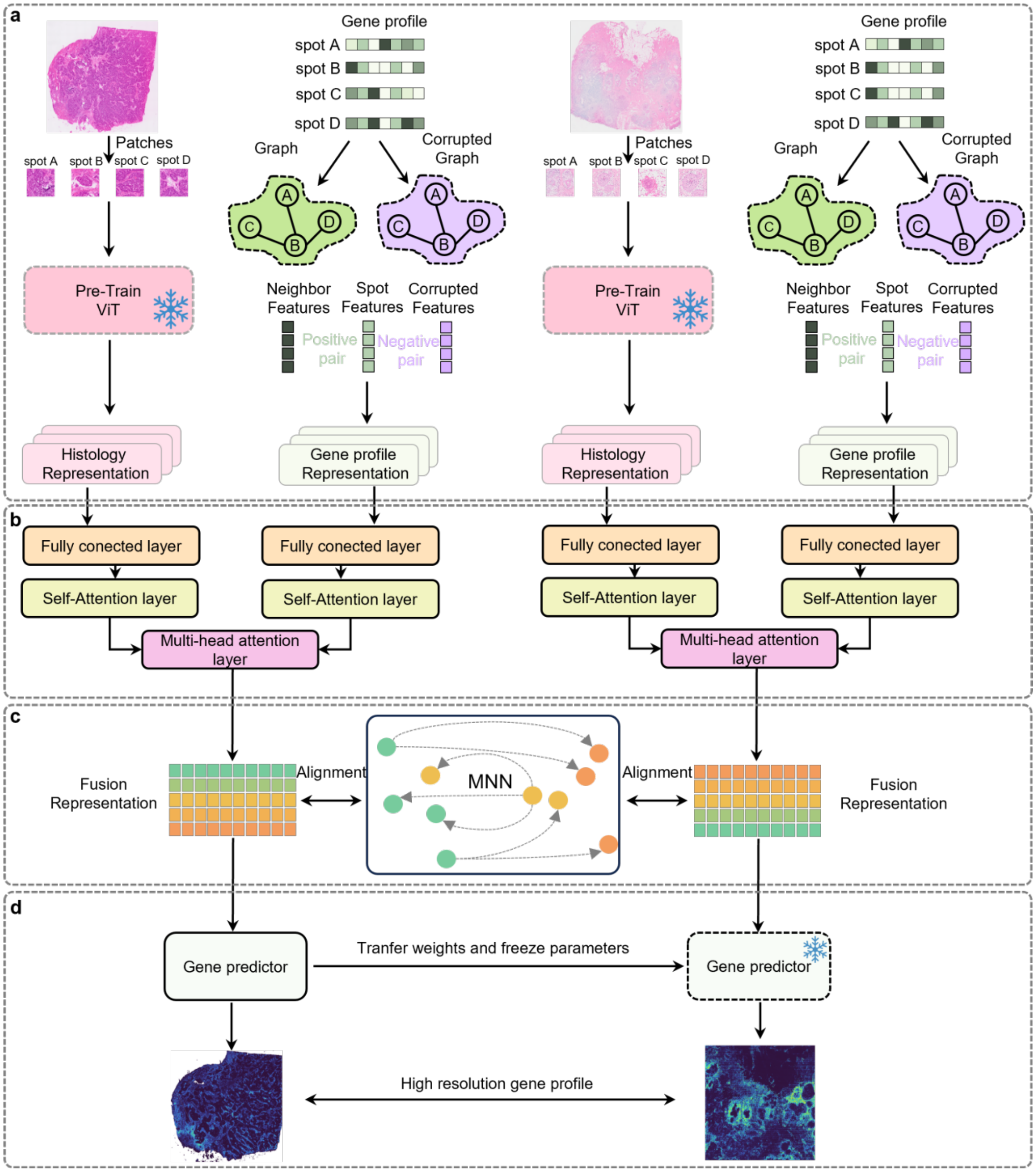

Supplementary Figure 1: SpotZoomer structure. (**a**) Feature extraction in SpotZoomer: For gene expression profiles, we use a graph comparison approach for feature extraction, and for histology images, we use a pathology model pre-trained on ViT (UNI). (**b**) Multimodal feature fusion in SpotZoomer: For multimodal features from histological images and gene expression profiles, we adopt a bidirectional multi-head attention mechanism. We first use the self-attention mechanism to calculate the attention scores between features under the same modality, and then use multi-head attention to fuse the attention scores between the two modalities to finally obtain the fused latent representation. (**c**) Aligning latent representations in SpotZoomer of different resolutions: In order to align the latent representations after fusion of different resolution platforms, we use MNN and triplet loss to align the latent representations from the low-resolution platform to the latent representations from the high-resolution platform. (**d**) High-resolution gene prediction in SpotZoomer :the prediction encoder is trained using HD-level supervised learning on the Visium HD platform and spot-level weak self-supervised learning on the Visium platform.

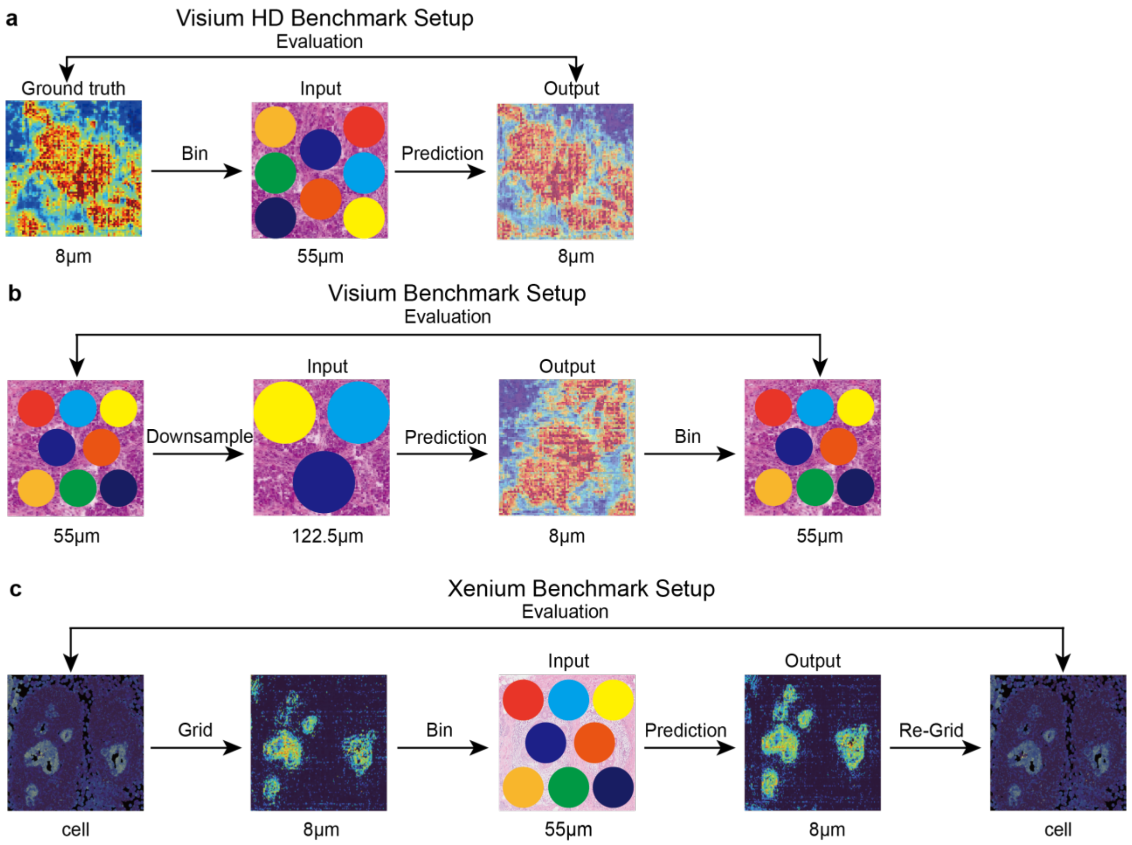

Supplementary Figure 2: **Benchmark setup in different platforms.** (**a**) Visium HD platform benchmark setup. For the Visium HD platform, we simulated pseudo data at the Visium level based on the HD level resolution as input, and the output super-resolution expression spectrum was compared with the original data for quantitative evaluation of the evaluation metrics. (**b**) Visium platform benchmark setup. For the Visium platform, we downsample the original Visium level data to obtain a sub Visium level low-resolution pseudo data as input. The super-resolution gene expression spectrum output by the model is downsampled to the Visium level data according to the coordinates, and then the evaluation metrics are calculated with the original data. (**c**) Xenium platform benchmark setup. For the Xenium platform, we first simulated a grid-based sequencing resolution using the cell boundary file provided by the platform. The irregular cell-level expression data were regularized to a grid format, from which we generated pseudo Visium-level input data. The model then produced outputs at a super-resolution scale. Finally, we applied a re-gridding process to map the predictions back to the original cell scale and computed the evaluation metrics accordingly.

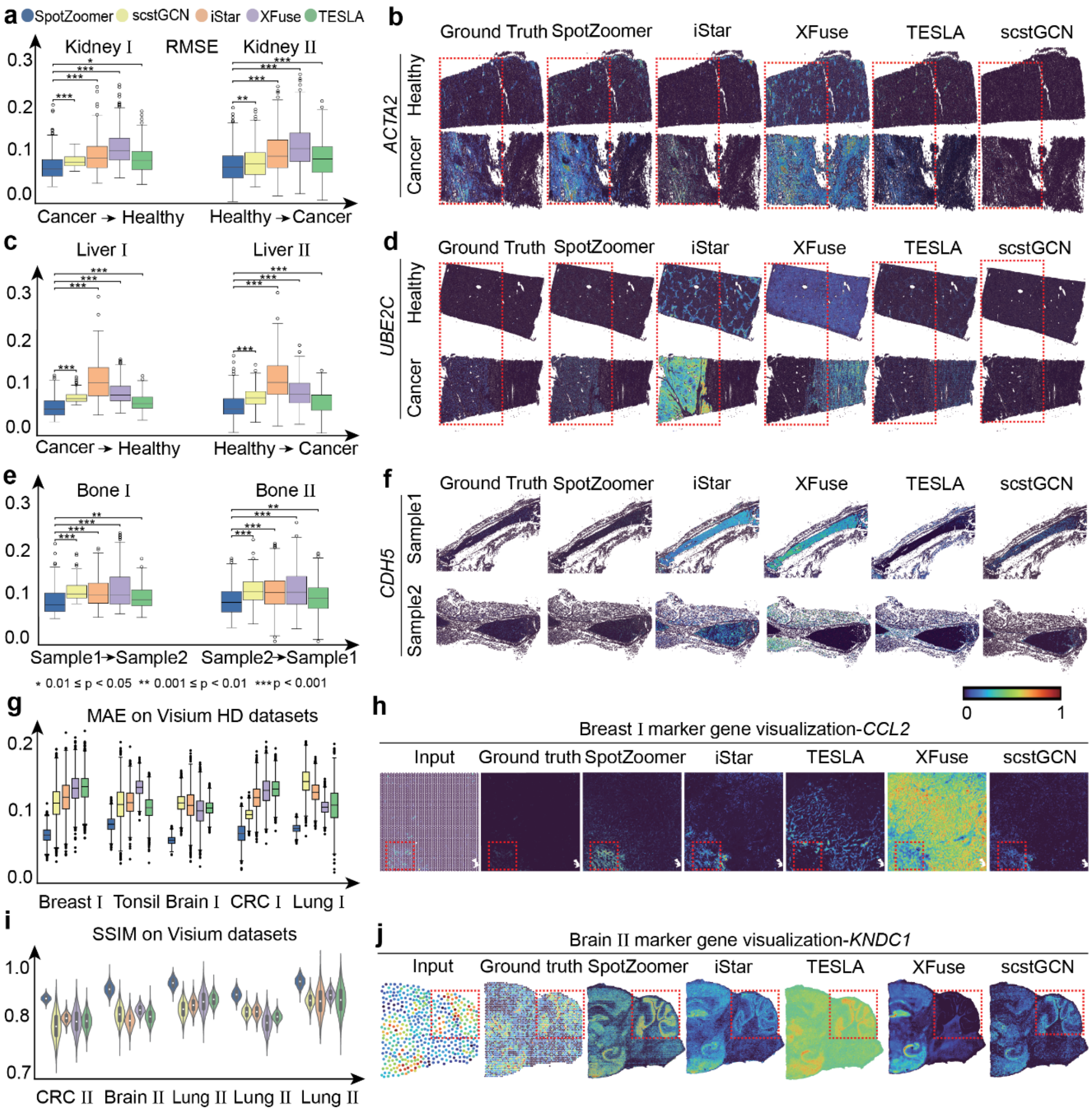

Supplementary Figure 3: **Evaluation of SpotZoomer on spatial transcriptomics datasets from multiple tissues and platforms. (a, c, e)** Performance evaluation using root mean square error (RMSE) on spatial datasets of Kidney, Liver and Bone coming from Xenium platform, box plots display the distribution of per-gene metric values: the center line indicates the median; the box spans the interquartile range (IQR, 25th–75th percentile); whiskers extend to 1.5 × IQR beyond the box; points outside the whiskers are shown as individual outliers. Pairwise significance brackets indicate paired Wilcoxon signed-rank tests of SpotZoomer against each baseline method: *** $p < 0.001$, ** $p < 0.01$, * $p < 0.05$. **(b, d, f)** Spatial visualization of gene expression patterns of marker genes (ACTA2, UBE2C and CDH5) on the Xenium datasets. **(g)** Performance evaluation using mean absolute error (MAE) on Visium HD datasets. The box plots show the distribution of generated super-resolution gene expression, with the center line indicating the median and the boxes representing the interquartile range (IQR). **(h)** Spatial visualization of gene expression patterns of marker CCL2. **(i)** Performance evaluation using structural similarity index (SSIM) on Visium datasets, with the center line indicating the median and the boxes

representing the interquartile range (IQR). All pairwise paired Wilcoxon signed-rank tests of SpotZoomer against each baseline method (iStar, scstGCN, TESLA, XFuse) yielded $p < 0.001$. **(j)** Spatial visualization of gene expression patterns of marker KNDC1.

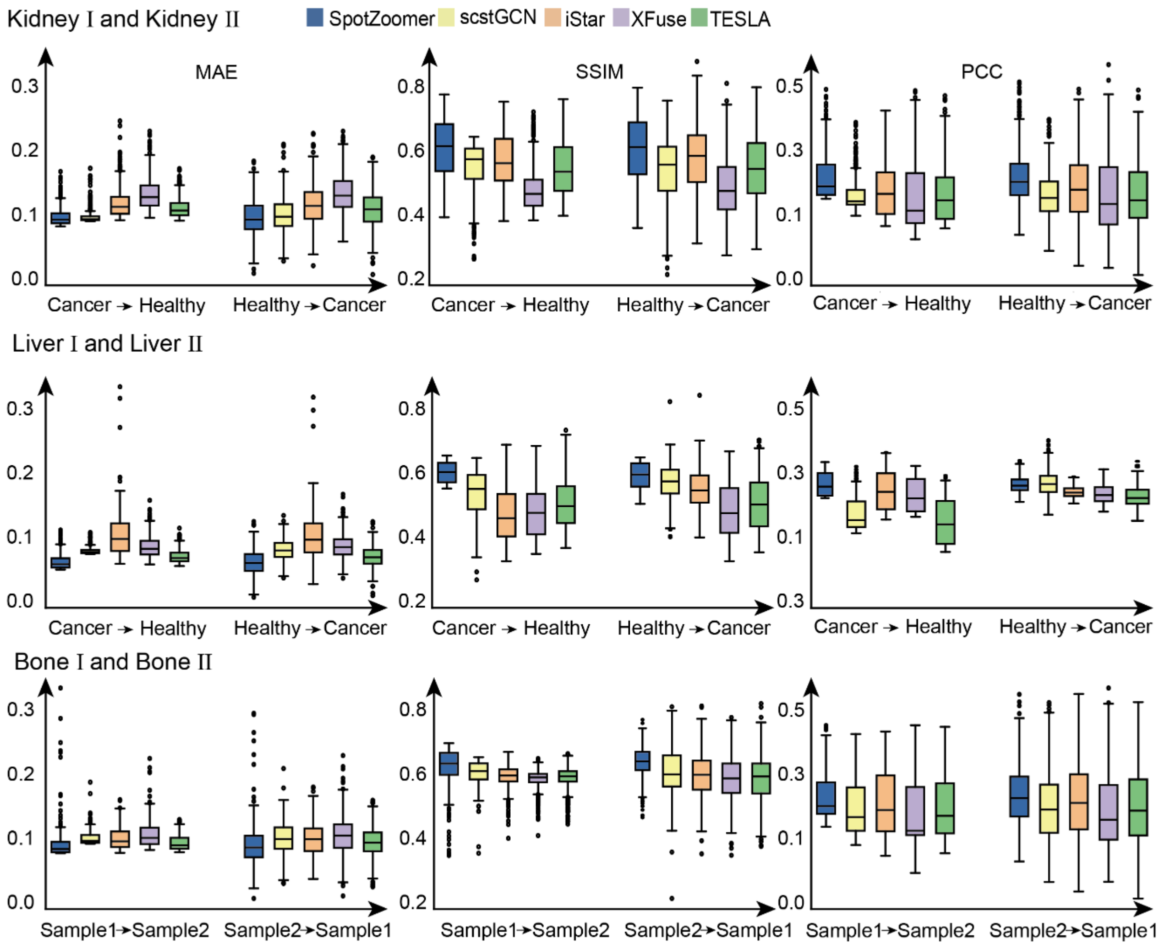

Supplementary Figure 4: **Evaluation of SpotZoomer on multiple spatial transcriptomics datasets from Xenium platform.** Box plots display the distribution of per-gene metric values: the center line indicates the median; the box spans the interquartile range (IQR, 25th–75th percentile); whiskers extend to 1.5 × IQR beyond the box; points outside the whiskers are shown as individual outliers.

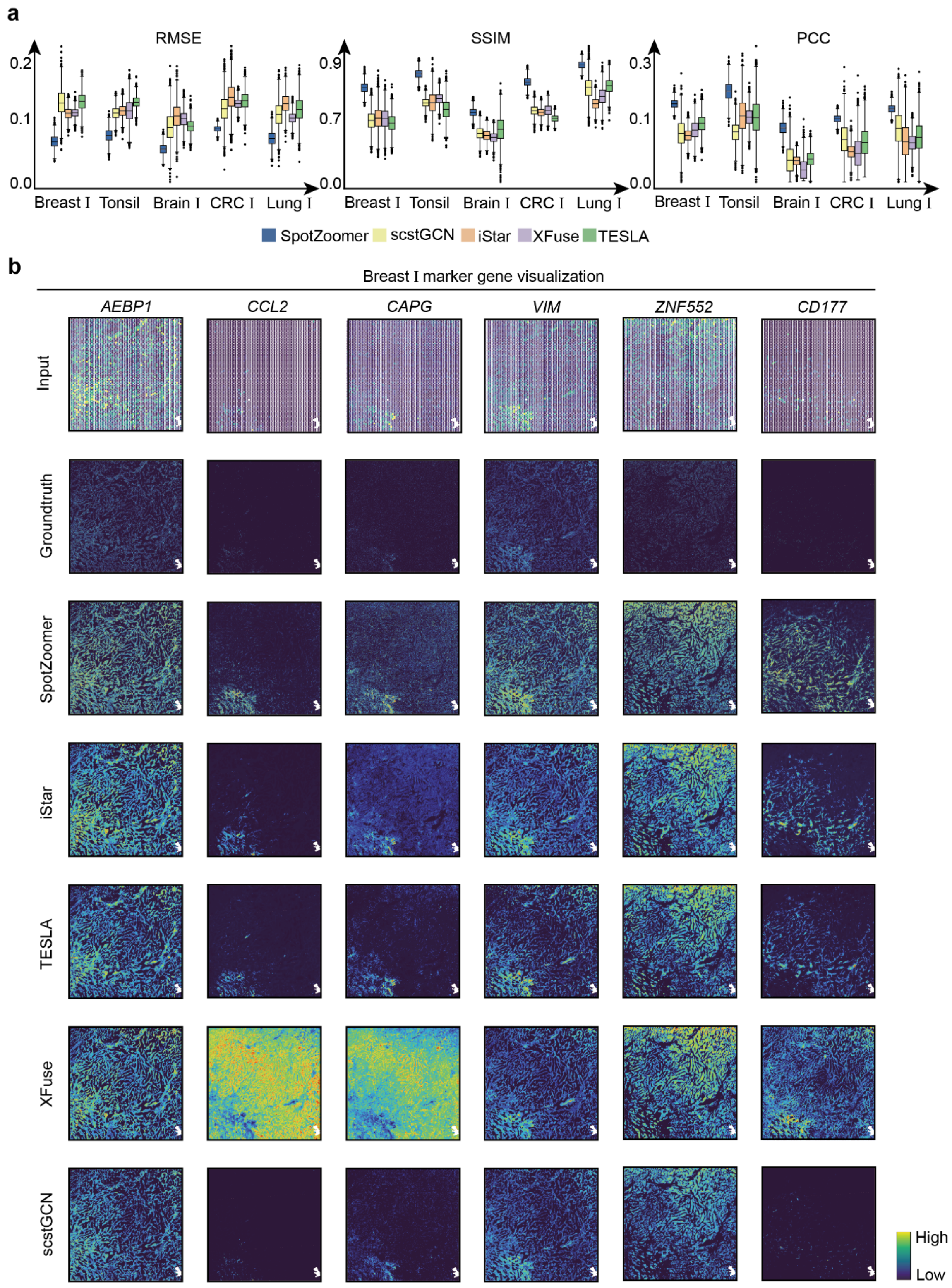

Supplementary Figure 5: **Evaluation of SpotZoomer on multiple spatial transcriptomics datasets from Visium HD platform and marker gene visualization.** (**a**) Model performance on multiple Visium HD datasets, evaluated using RMSE, SSIM, and PCC**,** box plots display the distribution of per-gene metric values: the center line indicates the median; the box spans the

interquartile range (IQR, 25th–75th percentile); whiskers extend to 1.5 × IQR beyond the box; points outside the whiskers are shown as individual outliers. (**b**) Marker gene visualization on Breast data.

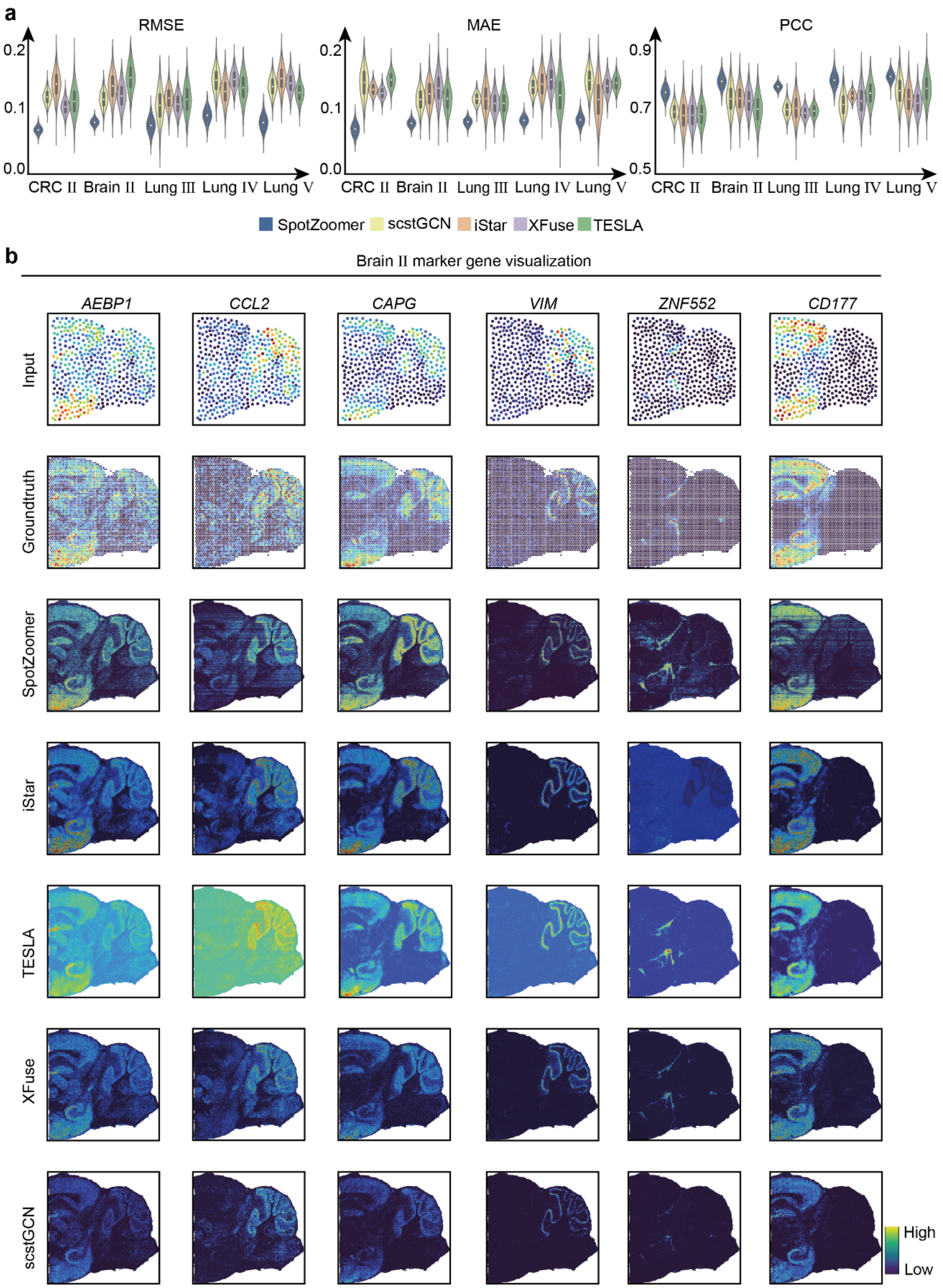

Supplementary Figure 6: **Evaluation of SpotZoomer on multiple spatial transcriptomics datasets from Visium platform and marker gene visualization.** (**a**) Model performance on multiple Visium HD datasets, evaluated using RMSE, MAE, and PCC. Violin plots display the kernel density estimate of per-gene metric values across all evaluated Visium datasets, mirrored

about the central axis to make the multimodal distributional structure visible. The inner box-and-whisker overlay follows the same convention as box plots elsewhere in this manuscript (center line: median; box: IQR; whiskers: 1.5 × IQR). All pairwise paired Wilcoxon signed-rank tests of SpotZoomer against each baseline method (iStar, scstGCN, TESLA, XFuse) yielded $p < 0.001$. (**b**) Marker gene visualization on Brain data.

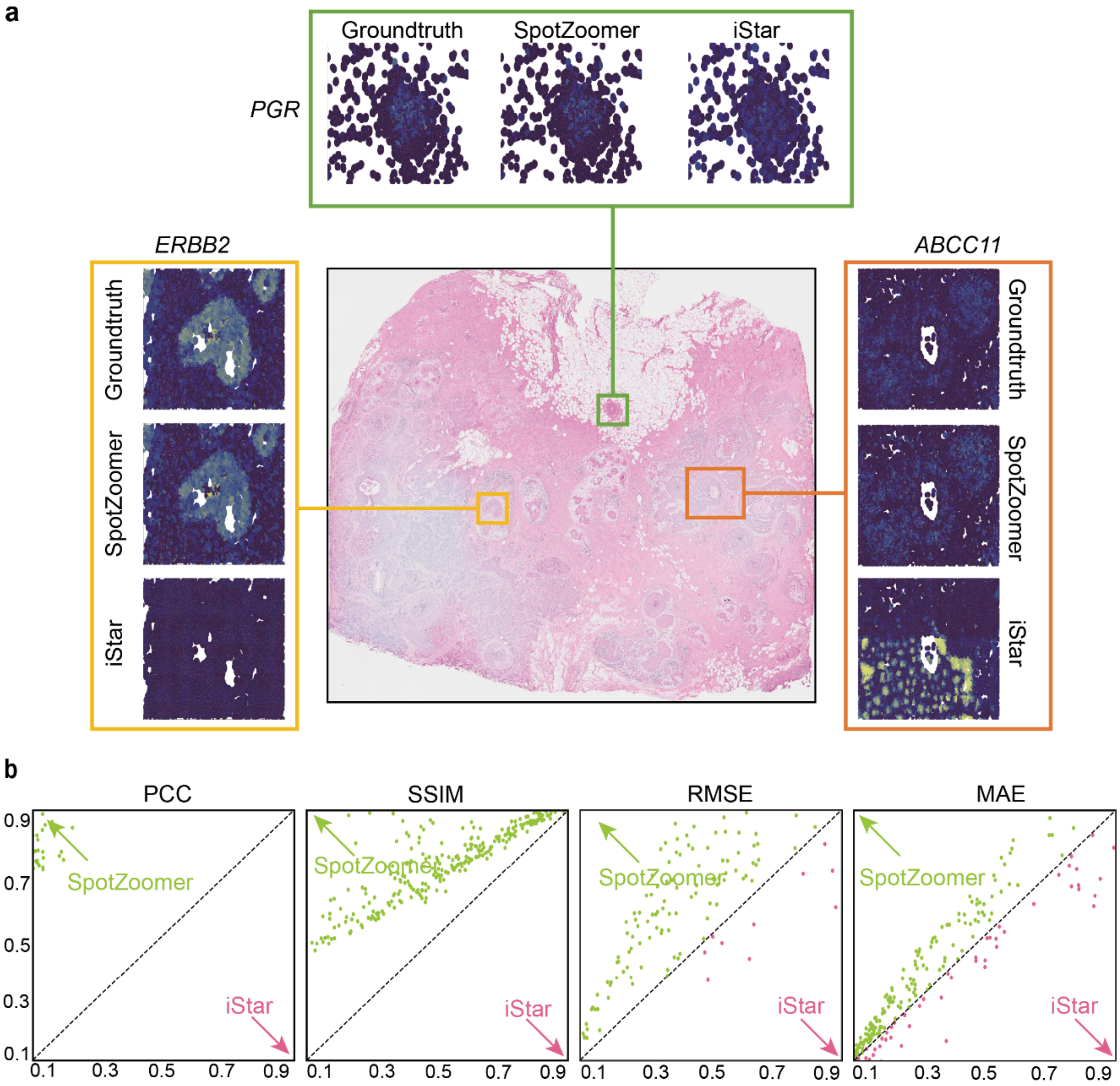

Supplementary Figure 7: **Visualization of single-cell level gene expression prediction and evaluation of prediction accuracy.** (**a**) Visualization of single-cell level gene expression predicted by SpotZoomer and iStar on Breast III. (**b**) Prediction accuracy of SpotZoomer and iStar on Breast data.

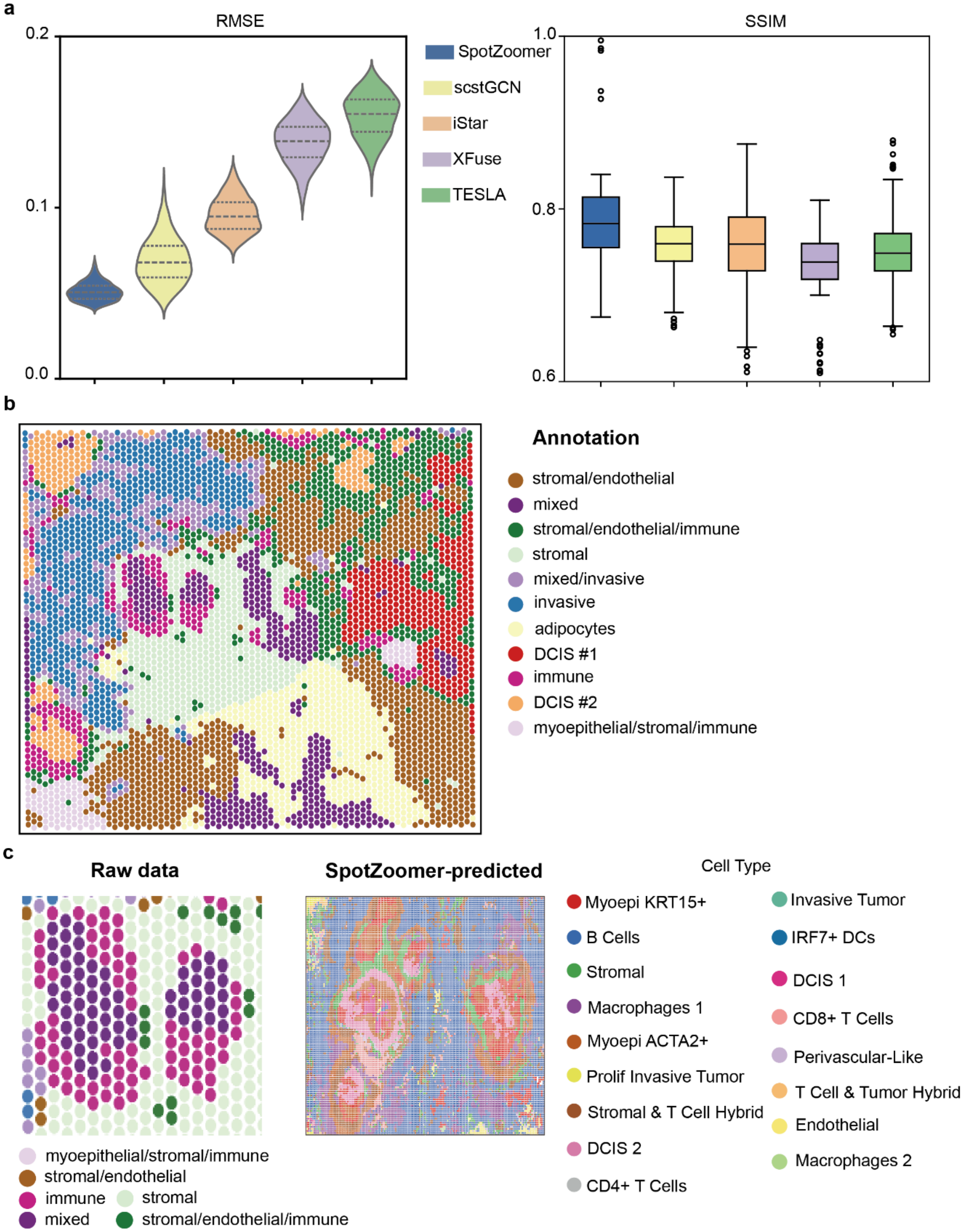

Supplementary Figure 8: **Super-resolution gene expression prediction accuracy on Breast II by different methods and its cell type annotation.** (**a**) Performance evaluation using SSIM and RMSE on Breast datasets. (**b**) Sectioning of Breast was performed according to the Visium CytAssist v2 WT Panel Gene Expression – Tissue Preparation Guide (Demonstrated Protocol CG000518), with cell type annotations included. (**c**) Cell types of the zoom-in region in the raw data and those predicted by SpotZoomer in TLS.

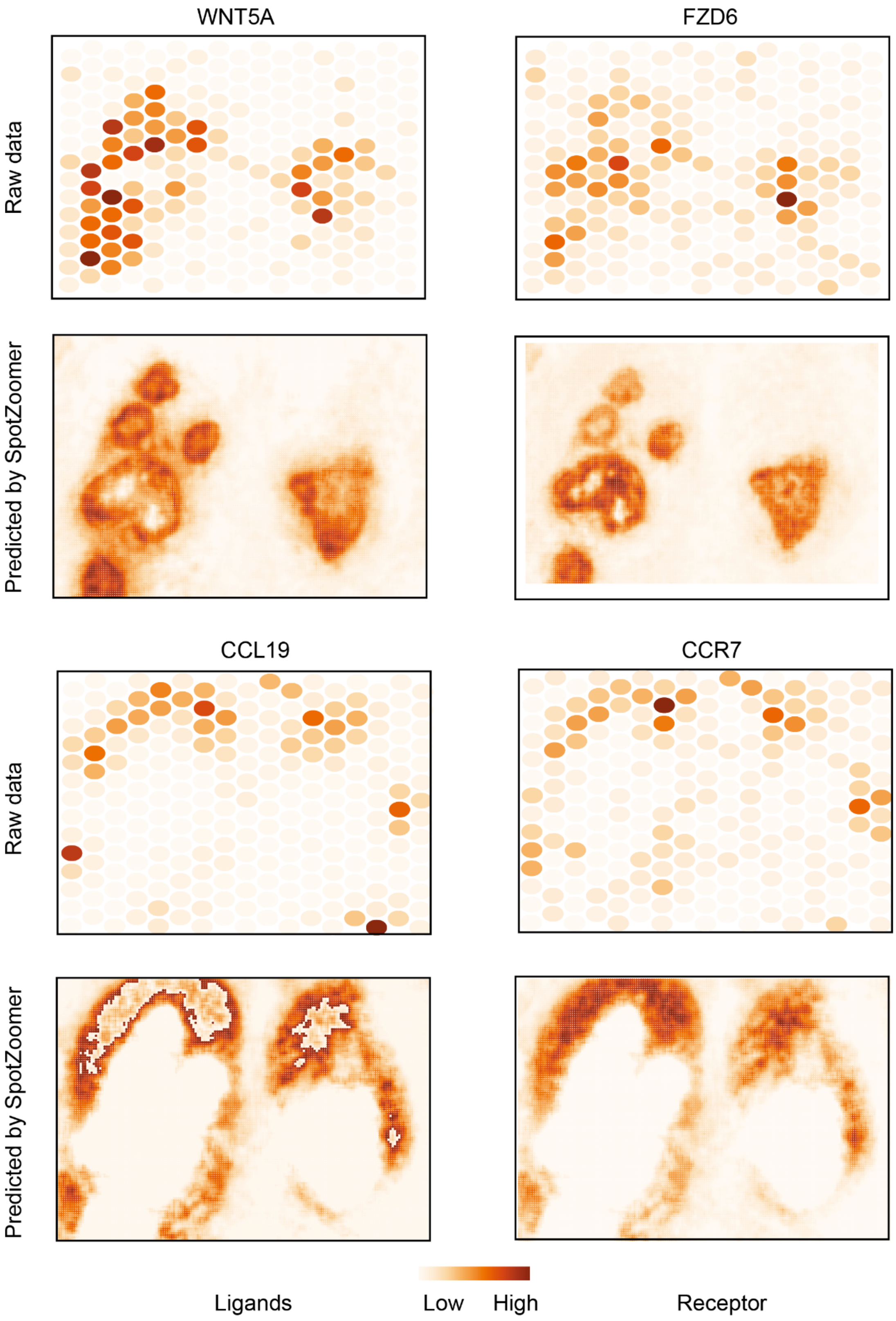

Supplementary Figure 9: Visual comparison of the communication intensities of the WNT5A–FZD6 and CCL19–CCR7 receptor-ligand pairs in raw data and high-resolution SpotZoomer predictions.

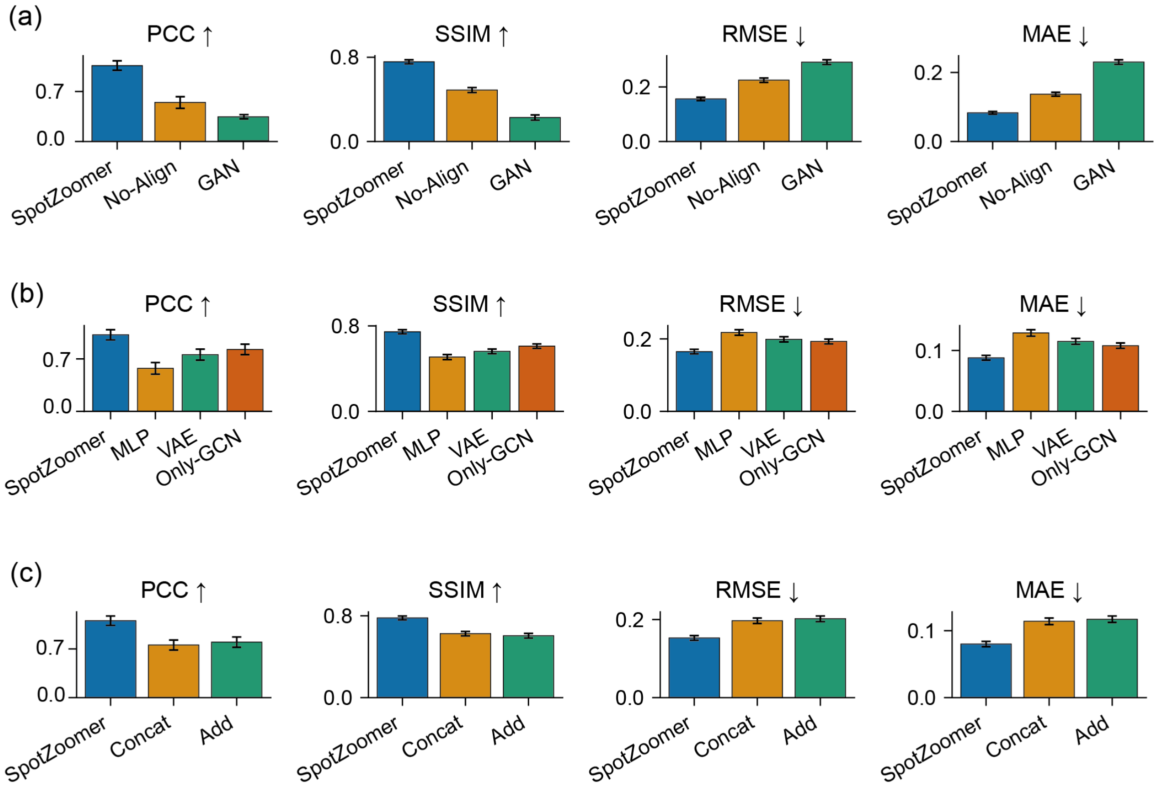

Supplementary Figure 10. Module-level ablation studies of SpotZoomer's core architectural components. (a) Cross-platform alignment ablation: default MNN + triplet loss compared against No-Align (alignment module removed) and GAN (adversarial domain adaptation). (b) Multimodal feature fusion ablation: default Bi-directional Multi-Head Attention (BMHA) compared against Concat (channel-wise concatenation + MLP projection) and Add (element-wise summation). (c) Graph contrastive learning ablation: default GCN + Contrastive Self-supervised Learning (GCN+CSL) compared against MLP (no graph), VAE (graph + ELBO objective) and Only-GCN (graph without contrastive objective).

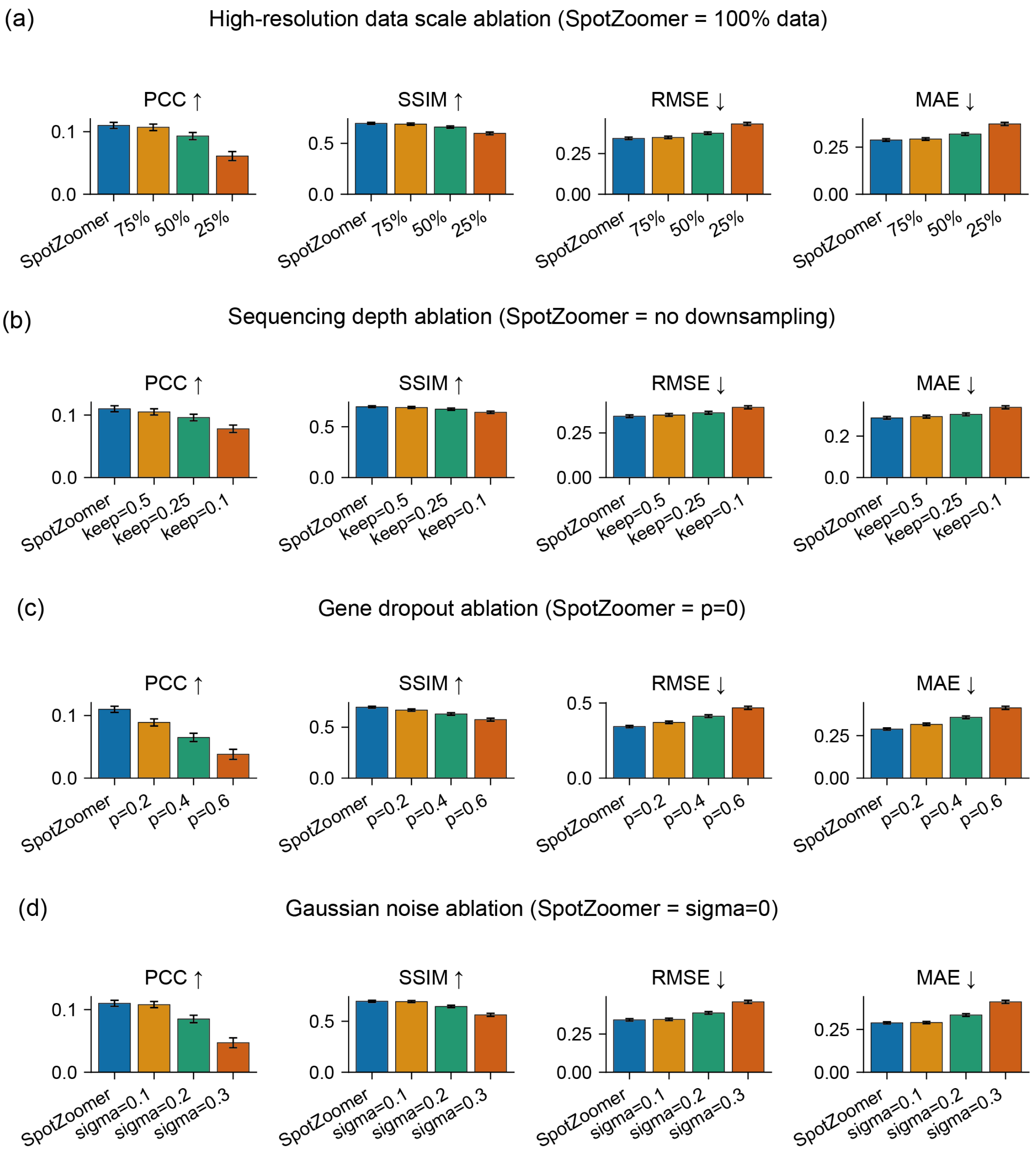

Supplementary Figure 11. Sensitivity of SpotZoomer to reference (Teacher) data quality and quantity. CRC I Section 1 (Visium HD, 8 μm) and CRC II (Visium, 55 μm) were used as Teacher and Student domains, respectively, following the benchmark protocol in Supplementary Fig. 2b. **(a)** Reference cohort size: Teacher spots subsampled to 100/75/50/25%. **(b)** Sequencing depth: binomial thinning of raw counts at retention probabilities $p \in \{1.0, 0.5, 0.25, 0.1\}$. **(c)** Capture efficiency: non-zero counts randomly set to zero at $p \in \{0, 0.2, 0.4, 0.6\}$. **(d)** Technical noise: additive Gaussian noise $N(0, \sigma)$ with $\sigma \in \{0, 0.1, 0.2, 0.3\}$ on min–max normalized counts.

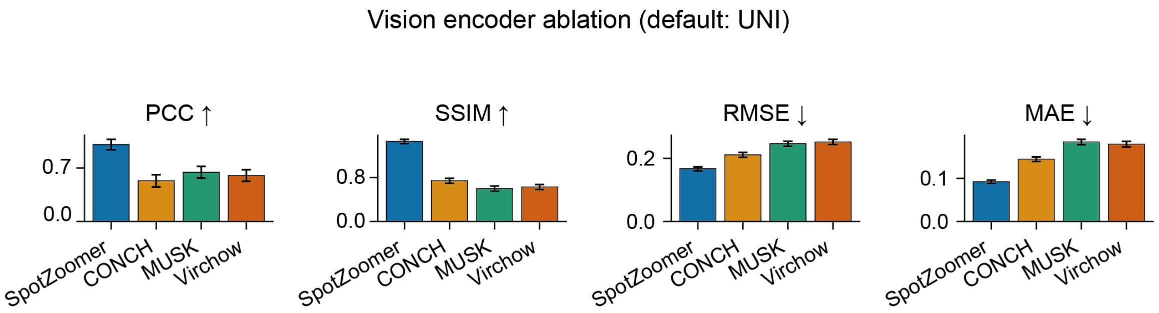

Supplementary Figure 12. Ablation of the H&E vision encoder. Comparison of SpotZoomer with the default UNI encoder against three alternative pathology foundation models (CONCH, MUSK, Virchow), with all other architectural components and training configurations held constant. Dataset configuration:. Bars show mean ± standard deviation across three random seeds; metrics: PCC, SSIM, RMSE, MAE.

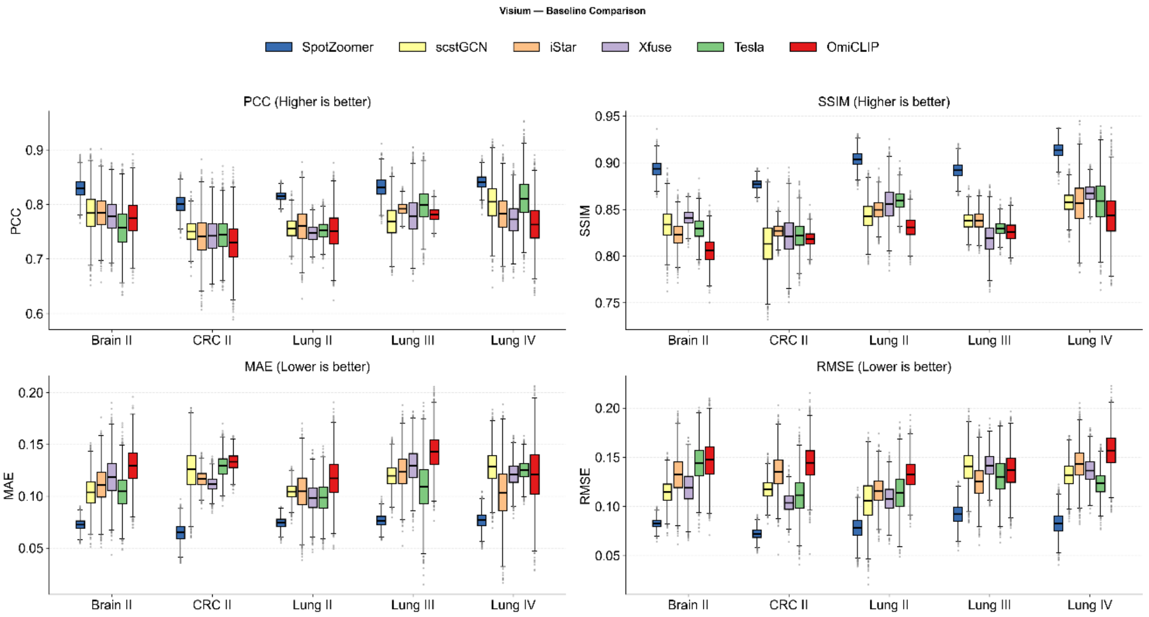

Supplementary Figure 13. Baseline comparison at Visium-spot resolution, OmiCLIP's native operating regime. SpotZoomer (blue) is compared against five baseline methods: scstGCN, iStar, XFuse, TESLA, and OmiCLIP on five Visium-platform datasets (Brain II, CRC II, Lung II, Lung III, Lung IV) across four reconstruction metrics: PCC (↑), SSIM (↑), MAE (↓), and RMSE (↓). SpotZoomer outperforms OmiCLIP on every metric across every dataset; OmiCLIP performs comparably to the other (non-SpotZoomer) baselines at this resolution, confirming that it operates as a competent vision–language model in its native regime. Box plots display per-gene metric distributions: center line, median; box, interquartile range (IQR, 25th–75th percentile); whiskers, 1.5 × IQR beyond the box; points beyond whiskers, individual outliers.

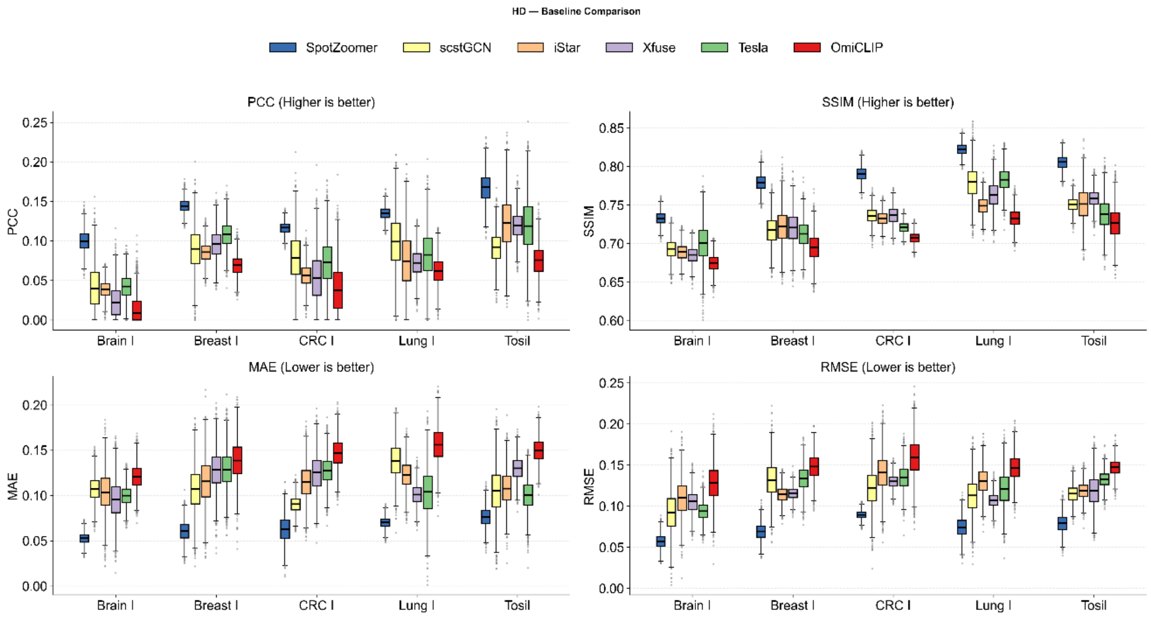

Supplementary Figure 14. Baseline comparison at high-resolution Visium HD / Xenium level, the super-resolution regime. SpotZoomer (blue) is compared against the same five baseline methods on five high-resolution datasets (Brain I, Breast I, CRC I, Lung I, Tonsil) across the same four reconstruction metrics. SpotZoomer leads on every metric across every dataset; OmiCLIP performs substantially worse than all super-resolution baselines (note in particular its near-zero median PCC on Brain I), consistent with its design as a native-resolution vision–language model rather than a super-resolution method. This empirical observation supports the manuscript's design choice to restrict its main benchmark to super-resolution methods only.

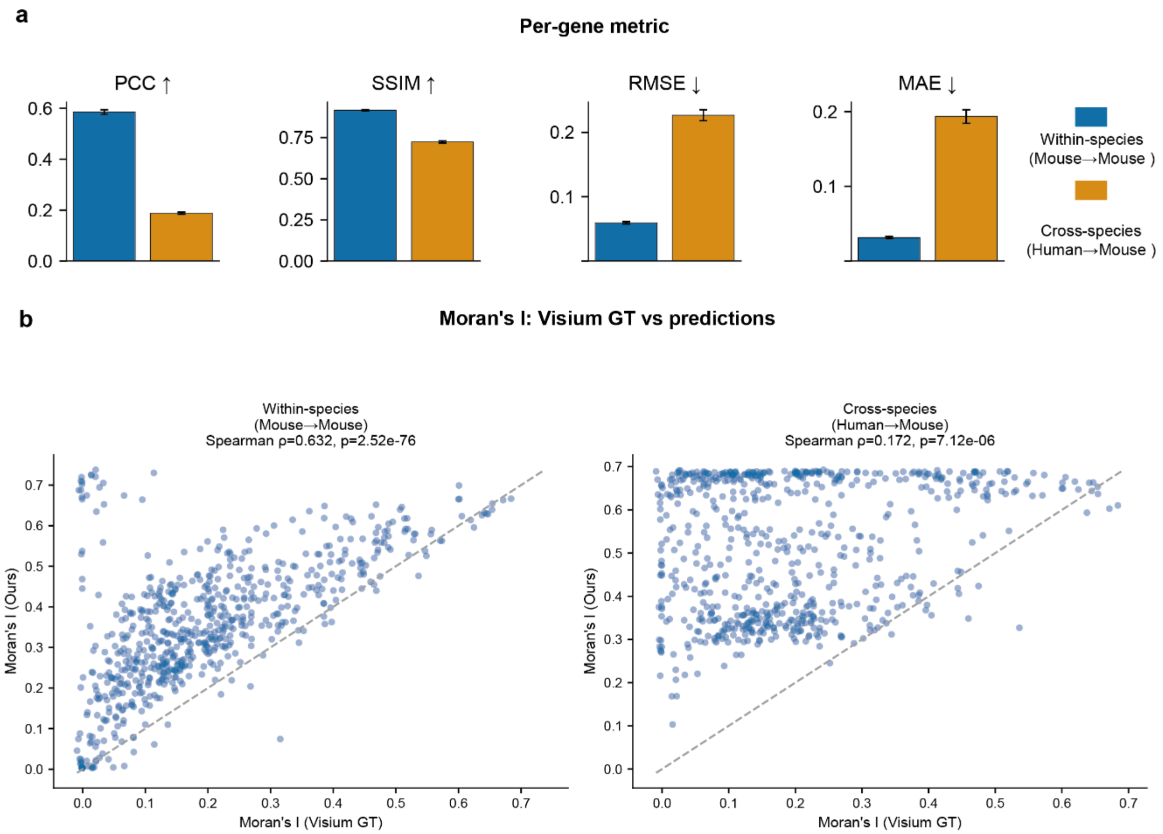

Supplementary Figure 15. Cross-species transferability of SpotZoomer. The same mouse lung Visium Student is used in both configurations; the Teacher is either within-species mouse lung Visium HD (blue) or cross-species human lung Visium HD (orange). (a) Per-gene reconstruction metrics (mean ± s.e.m.). Within-species transfer outperforms cross-species transfer on all four metrics—PCC ~0.58 vs ~0.19, SSIM ~0.91 vs ~0.72, RMSE ~0.06 vs ~0.23, and MAE ~0.04 vs ~0.19—indicating a consistent and substantial loss of per-gene reconstruction fidelity across species. (b) Per-gene Moran's I, ground truth (Visium) vs prediction. Within-species, predicted and ground-truth Moran's I are strongly and highly significantly correlated (Spearman $\rho = 0.632$, $p = 2.52 \times 10^{-76}$); under cross-species transfer the correlation remains positive and significant but is markedly weaker ($\rho = 0.172$, $p = 7.12 \times 10^{-6}$), with predictions biased toward high Moran's I values. Dashed line, identity.

Supplementary Table 1. Dataset for SpotZoomer training and evaluation

Dataset	Species	Tissue	Protocol	Unit	Section	Spot/cell	Gene
Breast I	Human	Breast Cancer	Visium HD	8μm	2	Section1: 472859<br>Section2: 700193	18085
Tosil	Human	Tosil	Visium HD	8μm	2	Section1: 541968<br>Section2: 545613	19059
Brain I	Mouse	Brain	Visium HD	8μm	2	Section1: 393543<br>Section2: 453541	18085
CRC I	Human	Colorectal Cancer	Visium HD	8μm	2	Section1: 656382<br>Section2: 764763	18085
Lung I	Human	Lung	Visium HD	8μm	2	Section1: 548683<br>Section2: 632641	18085
Lung II	Mouse	Lung	Visium HD	8μm	1	576389	18743
Breast II	Human	Breast Cancer	Visium	55μm	1	5712	40003
Brain II	Mouse	Brain	Visium	55μm	1	4912	32741
Lung III	Mouse	Healthy Lung	Visium	55μm	1	3937	20012
Lung IV	Mouse	Pulmonary fibrosis	Visium	55μm	1	4388	20012
Lung V	Mouse	Pulmonary fibrosis	Visium	55μm	1	4637	20012
CRC II	Human	Colorectal Cancer	Visium	55μm	1	5421	18671
Breast III	Human	Breast Cancer	Xenium	cell	1	118752	364
Kidney I	Human	Healthy Kidney	Xenium	cell	1	2730	541
Kidney II	Human	Kidney Cancer	Xenium	cell	1	1738	541
Liver I	Human	Healthy Liver	Xenium	cell	1	9200	541
Liver II	Human	Liver Cancer	Xenium	cell	1	4004	538
Bone I	Mouse	Bone marrow	Xenium	cell	1	9200	541

Bone II	Mouse	Bone marrow	Xenium	cell	1	12535	541

Supplementary Table 2. Analysis the dataset used in the experiment.

Experiment	Dataset
Visium HD experiment in Breast I	High resolution: Breast I, Section1; Low resolution: Breast I, Section2
Visium HD experiment in Tonsil	High resolution: Tonsil, Section1; Low resolution: Tonsil, Section2
Visium HD experiment in Brain I	High resolution: Brain I, Section1; Low resolution: Brain I, Section2
Visium HD experiment in CRC I	High resolution: CRC I, Section1; Low resolution: CRC I, Section2
Visium HD experiment in Lung I	High resolution: Lung I, Section1; Low resolution: Lung I, Section2
Xenium experiment in Kidney I	High resolution: Kidney II; Low resolution: Kidney I
Xenium experiment in Kidney II	High resolution: Kidney I; Low resolution: Kidney II
Xenium experiment in Liver I	High resolution: Liver II; Low resolution: Liver I
Xenium experiment in Liver II	High resolution: Liver I; Low resolution: Liver II
Xenium experiment in Bone I	High resolution: Bone II; Low resolution: Bone I
Xenium experiment in Bone II	High resolution: Bone I; Low resolution: Bone II
Visium experiment in CRC II	High resolution: CRC I, Section1; Low resolution: CRC II
Visium experiment in Brain II	High resolution: Brain I, Section1; Low resolution: Brain II
Visium experiment in Lung III	High resolution: Lung II; Low resolution: Lung III
Visium experiment in Lung IV	High resolution: Lung II; Low resolution: Lung IV
Visium experiment in Lung V	High resolution: Lung II; Low resolution: Lung V
CRC analysis of SpotZoomer	High resolution: CRC I, Section1; Low resolution: CRC II
HBC analysis of SpotZoomer	High resolution: Breast I, Section1; Low resolution: Breast II
pulmonary fibrosis of SpotZoomer	High resolution: Lung II; Low resolution: Lung III, Lung IV, Lung V

Supplementary Table 3. TLS marker genes.

TLS Markers
CD4
CD8A
CD74
CD79A
IL7R
ITGAE
CD1D
CD3D
CD3E
CD8B
CD19
CD22
CD52
CD79B
CR2
CXCL13
CXCR5
FCER2
MS4A1
PDCD1
PTGDS
TRBC2

Supplementary Table 4. Runtime and memory consuming of SpotZoomer on different sequencing platforms. Computational cost comparison of SpotZoomer and baseline methods (iStar, XFuse, TESLA, scstGCN) across two representative platform configurations (Visium HD to Visium and Xenium to Visium). Reported metrics include end-to-end runtime (seconds), peak CPU memory consumption (MB), and peak GPU memory consumption (MB). All values represent the mean across all 18 evaluated datasets spanning multiple tissue types and species (breast, brain, kidney, liver, lung, bone, colorectal cancer, tonsil). XFuse is not GPU-accelerated and therefore has no GPU memory entry.

VisiumHD-Visium	SpotZoomer	iStar	XFuse	TESLA	scstGCN
Time / Seconds	341	241	48367	90247	287
CPU memory Usage / MB	6624	6781	*4863*	17043	7521
GPU memory Usage / MB	3014	2776	6049	-	2561
Xenium-Visium	SpotZoomer	iStar	XFuse	TESLA	scstGCN
Time / Seconds	297	227	39671	86299	208
CPU memory Usage / MB	6408	6125	4771	15863	7412
GPU memory Usage / MB	3677	2561	5889	-	2477

Supplementary Table 5. Complete hyperparameter settings for all SpotZoomer experiments reported in this manuscript.

Category	Hyperparameter	Value
**Optimizer**		
	Optimizer	AdamW
	(β_1, β_2, ε)	(0.9, 0.999, 1×10^{-8})
	Initial learning rate	1×10^{-3}
	Final learning rate (after cosine decay)	1×10^{-5}
	LR scheduler	Cosine annealing
	Weight decay	1×10^{-4}
	Gradient clipping (global L2 norm)	1.0
**Batch / Training**		
	Batch size	1024 spots per gradient step (mini-batch, sampled without replacement within each epoch)
	Random seed (main)	42
	Additional seeds (sensitivity check)	0, 1, 2
**Loss weights**		
	λ_1 (alignment / triplet loss)	0.7
	λ_2 (contrastive self-supervision)	1.2
	λ_3 (reconstruction / prediction)	1
	Triplet margin δ	0.5
	Smoothness coefficient α	1.0
**Architecture — Gene encoder**		

Category	Hyperparameter	Value
	GCN layers	3
	GCN hidden dimension	256
	Output embedding dimension	256
**Architecture — H&E encoder**		
	Backbone	UNI (frozen, loaded from Hugging Face)
	Output embedding dimension	256
**Architecture — Fusion (BMHA)**		
	Attention heads	8
	Hidden dimension	256
	Number of fusion layers	3
**Architecture — Predictor**		
	Predictor MLP hidden dimension	256
	Predictor MLP depth	3 layers
	Frozen layers during fine-tune phase	All except final layer
**Multi-stage training schedule**		
	Phase 1 (encoder + fusion)	$0 < t \leq 0.2$ E
	Phase 2 (predictor pre-train on pseudo-LR)	0.2 E $< t \leq 0.5$ E
	Phase 3 (predictor fine-tune on Student)	0.5 E $< t \leq$ E
**Hardware**		
	GPU	NVIDIA A100 80G
	CUDA / PyTorch versions	CUDA 12.8/ PyTorch 2
